# Neuronal ELAVL proteins utilize AUF-1 as a co-partner to induce neuron-specific alternative splicing of APP

**DOI:** 10.1038/srep44507

**Published:** 2017-03-14

**Authors:** Apostolia Fragkouli, Pelagia Koukouraki, Ioannis S. Vlachos, Maria D. Paraskevopoulou, Artemis G. Hatzigeorgiou, Epaminondas Doxakis

**Affiliations:** 1 Center for Basic Research, Biomedical Research Foundation Academy of Athens, 4 Soranou Efesiou str, 11527, Athens Greece; 2 Hellenic Pasteur Institute, 127 Vasilissis Sofias Avenue, 11521 Athens, Greece; 3 DIANA-Lab, Department of Electrical & Computer Engineering, University of Thessaly, 38221 Volos, Greece

## Abstract

Aβ peptide that accumulates in Alzheimer’s disease brain, derives from proteolytic processing of the amyloid precursor protein (APP) that exists in three main isoforms derived by alternative splicing. The isoform APP695, lacking exons 7 and 8, is predominately expressed in neurons and abnormal neuronal splicing of *APP* has been observed in the brain of patients with Alzheimer’s disease. Herein, we demonstrate that expression of the neuronal members of the ELAVL protein family (nELAVLs) correlate with APP695 levels *in vitro* and *in vivo*. Moreover, we provide evidence that nELAVLs regulate the production of APP695; by using a series of reporters we show that concurrent binding of nELAVLs to sequences located both upstream and downstream of exon 7 is required for its skipping, whereas nELAVL-binding to a highly conserved U-rich sequence upstream of exon 8, is sufficient for its exclusion. Finally, we report that nELAVLs block APP exon 7 or 8 definition by reducing the binding of the essential splicing factor U2AF65, an effect facilitated by the concurrent binding of AUF-1. Our study provides new insights into the regulation of APP pre-mRNA processing, supports the role for nELAVLs as neuron-specific splicing regulators and reveals a novel function of AUF1 in alternative splicing.

One of the hallmarks of Alzheimer’s disease (AD), the major cause of dementia in humans, is the abnormal deposition of aggregated amyloid-beta peptide (Αβ) in the brain^1^. Aβ is generated upon proteolytic cleavage of the parent amyloid precursor protein (APP), mutations of which have been associated with familial AD^2^. In mammals, APP is ubiquitously expressed, but most abundantly in the brain^3^,^4^. The gene contains 18 exons, four of which undergo alternative splicing (AS)^3^,^5^,^6^,^7^,^8^. In neurons, APP is present in three major isoforms: APP770 containing all exons, APP751 lacking exon 8 and APP695 lacking exons 7 and 8. APP770 and APP751 are ubiquitously expressed, whereas APP695 is predominately enriched in neurons^3^,^6^,^7^. It has been shown that APP695 is decreased, whereas APP770 is increased in the brain of AD patients^9^,^10^,^11^,^12^ and abnormal neuronal splicing of APP pre-mRNA has been associated with aberrant Aβ production^13^,^14^.

Along the same lines, AS is particularly prevalent in the nervous system^15^,^16^ and alterations in neural AS patterns have been linked to neurological disorders^17^. Tissue specific AS is regulated by a subset of RNA-binding proteins (RBPs), namely splicing regulators, that interact with RNA motifs on their target pre-mRNAs and affect their processing. In mammals, a highly abundant family of RBPs is the ELAVL-like (ELAVL) group of proteins that share significant homology with the *Drosophila* embryonic lethal and abnormal vision (ELAV) protein, shown to regulate AS in *Drosophila* neurons^18^,^19^. ELAVL protein family is comprised of four highly conserved members, the ubiquitously expressed ELAVL1 and the three predominantly neuronal RBPs ELAVL2, 3 and 4, collectively referred as neuronal ELAVL proteins (nELAVLs)^20^. At the molecular level, all members consist of three RNA-recognition motifs (RRM1-3) and a highly variable hinge region separating RRM2 and RRM3; RRM1 and RRM2 are mainly responsible for RNA-binding, whereas the hinge region is responsible for protein-protein interactions and contains signals for both nuclear localization and export^21^,^22^,^23^,^24^.

ELAVLs have been implicated in various biological processes and most of their functions were initially attributed to their ability to bind to specific AU-rich sequences (AU-rich elements, AREs) in the 3′untranslated region (UTR) of mammalian transcripts and increase the stability and/or translation of their targets^25^. More recent findings, suggest that at least nELAVLs bind with a higher affinity to U-rich sequences interspersed with guanosine (G) rather than adenosine residues (A), that are highly represented in intronic regions of target transcripts^26^. Consistently, in addition to the regulation of steady-state mRNA levels, nELAVLs have been also implicated in neuron-specific alternative RNA processing^26^ and nELAVL-mediated regulation of neuron-specific AS has been demonstrated for a small number of pre-mRNA targets^27^,^28^,^29^,^30^.

It has been previously shown that ELAVL4 binds to the 3′UTR of *APP* mRNA and increases its stability^31^. In the present study we provide evidence that nELAVLs are also critically involved in the regulation of neuron-specific AS of both human and mouse APP pre-mRNA. We report that APP695 expression correlates with nELAVLs in the human brain and cell lines and most importantly demonstrate that nELAVs but not the ubiquitous ELAVL1, promote the exclusion of both exons 7 and 8 from the pre-mRNA to generate APP695. Moreover, by using a series of artificial minigenes for human and mouse APP exons 7 and 8, we show that concurrent binding of nELAVLs to distal sequences located both upstream and downstream of exon 7 is required for its skipping, whereas nELAVL-binding to a highly conserved U-rich sequence just upstream of exon 8, is sufficient for its exclusion. Finally, we report that nELAVL proteins block exon definition by reducing the binding of the essential splicing factor U2AF65 to the 3′ splice site of both APP exons 7 and 8 and that this effect is facilitated by the concurrent binding of another ARE-binding protein AUF-1 to *APP* pre-mRNA. Taken together, our study provides new insights into a conserved neuronal mechanism for the regulation of APP695 expression and reveals a novel function of AUF-1 as an AS modulator.

## Results

### Correlation between nELAVLs and APP695 expression

**Figure 1:**
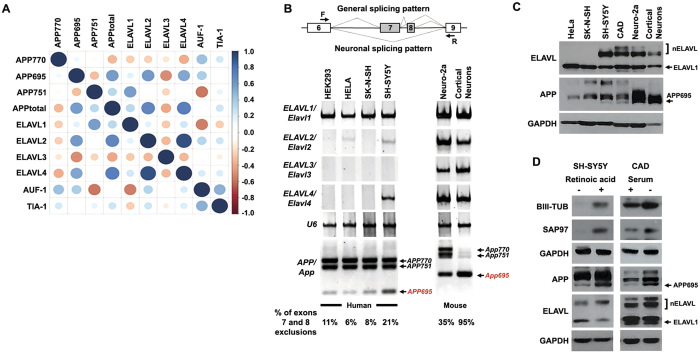
Expression of nELAVLs correlates with the APP695-specific pre-mRNA processing. (**A**) High-throughput sequencing data from 17 human samples were analyzed for the association between *APP* isoforms and *Hu, AUF*-*1* and *TIA*-*1* mRNA expression. Color intensity and circle size indicate the strength of the correlation. Note that only APP695 is correlated with nELAVL expression. (**B**) Schematic representation of APP AS in neuronal and non-neuronal cells and localization of primers used in this study (*arrows*, F: forward, R: reverse) Boxes indicate exons and bold lines introns. RT-PCR was carried out using total RNA isolated from five cell lines and primary cortical neurons with primers specific for human or mouse ELAVL1, ELAVL2, ELAVL3, ELAVL4, and primers for human or mouse APP that allow the simultaneous detection of all AS events of exons 7 and 8. The indicated amplification bands resulting from AS of APP exons 7 and/or 8 were identified by their respective length. Quantification of the results was performed by scanning densitometry and the percentage of exclusion of both exons 7 and 8 is indicated below each lane. (**C**) Equal amounts of total protein from lysates of five cell lines and cortical neurons were analyzed on SDS-PAGE and immunoblotted with antibodies specific for ELAVLs, APP and GAPDH as a loading control. Note that similar to cortical neurons, significant levels of APP695 were observed only in the cells lines expressing nELAVLs. (**D**) Human SH-SY5Y and mouse CAD neuroblastoma cells were differentiated into a neuronal-like phenotype. Equal amounts of total protein from lysates of the above untreated and differentiated cells were analyzed on SDS-PAGE and immunoblotted with antibodies specific for the neuronal markers β-ΙΙΙ tubulin and SAP97 as well as for APP, ELAVLs and finally GAPDH as a loading control. Note that differentiated SH-SY5Y and CAD cells displayed a concurrent upregulation of nELAVLs and APP695.

Both APP695 and nELAVLs are predominantly expressed in neurons^3^,^20^ indicating that a possible correlation between them may exist *in vivo*. To investigate this, we re-analyzed RNAseq data from 17 post-mortem human brain samples (9 AD patient and 8 control post-mortem brain samples; accession number GSE53699)^32^. More specifically, mRNA levels of isoforms *APP770, APP751* and *APP695* were normalized against total *APP* in each sample and associations between normalized *APP* isoform levels and mRNA levels of *ELAVL1*-*4*, as well as *AUF*-*1* and *TIA*-*1*, two other RBPs sharing common targets with ELAVLs^27^,^29^,^33^,^34^, were investigated (Fig. 1A). Our analysis revealed a significant correlation of neuronal *APP695* with *nELAVLs (ELAVL2*: Pearson r = 0.593, P < 0.05, *ELAVL3*: Pearson r = −0.510, P < 0.05, *ELAVL4*: Pearson r = 0.544, P < 0.05), but not with *ELAV1, AUF*-*1 or TIA*-*1*. Moreover, there was no correlation between the levels of *nELAVLs* and *APP770* or *APP751* isoforms (see Supplementary Table S1A,B) suggesting that in the human brain, *nELAVL* expression is specifically correlated with *APP695* expression. It should be noted that unlike previous reports^9^,^10^,^11^,^12^ our differential expression analysis revealed no difference in the levels of *APP* isoforms between control and AD samples. No difference was either observed in *nELAVL* levels between the two groups.

To examine if this correlation also applies *in vitro*, levels of ELAVLs and APP isoforms were assayed in different cell lines and mouse primary cortical neurons, the later serving as a cell model for APP695-specific AS. As shown in Fig. 1B and C, similar to cortical neurons, significant levels of APP695 mRNA and protein were detected only in cells, where nELAVL expression was also observed. In contrast, there was no obvious association between APP695 and ELAVL1 expression (Fig. 1B and C) or between nELAVLs and APP isoforms generated by AS of exon 15 (see Supplementary Fig. S1A–C). To gain further support, nELAVL and APP695 levels were also examined in SH-SY5Y and CAD neuroblastoma cells upon differentiation into a neuron-like phenotype. Compared to their controls, differentiated SH-SY5Y and CAD cells, as evident by the upregulation of neuronal markers βΙΙΙ tubulin and SAP97, displayed an increase of both nELAVLs and APP695 levels, whereas changes in ELAVL1 expression did not correspond to those of APP695 (Fig. 1D). It thus appears that a strong correlation between nELAVLs and APP695 generation exists both *in vitro* and *in viv*o.

### nELAVLs promote the exclusion of exons 7 and 8 from the endogenous APP pre-mRNA

**Figure 2:**
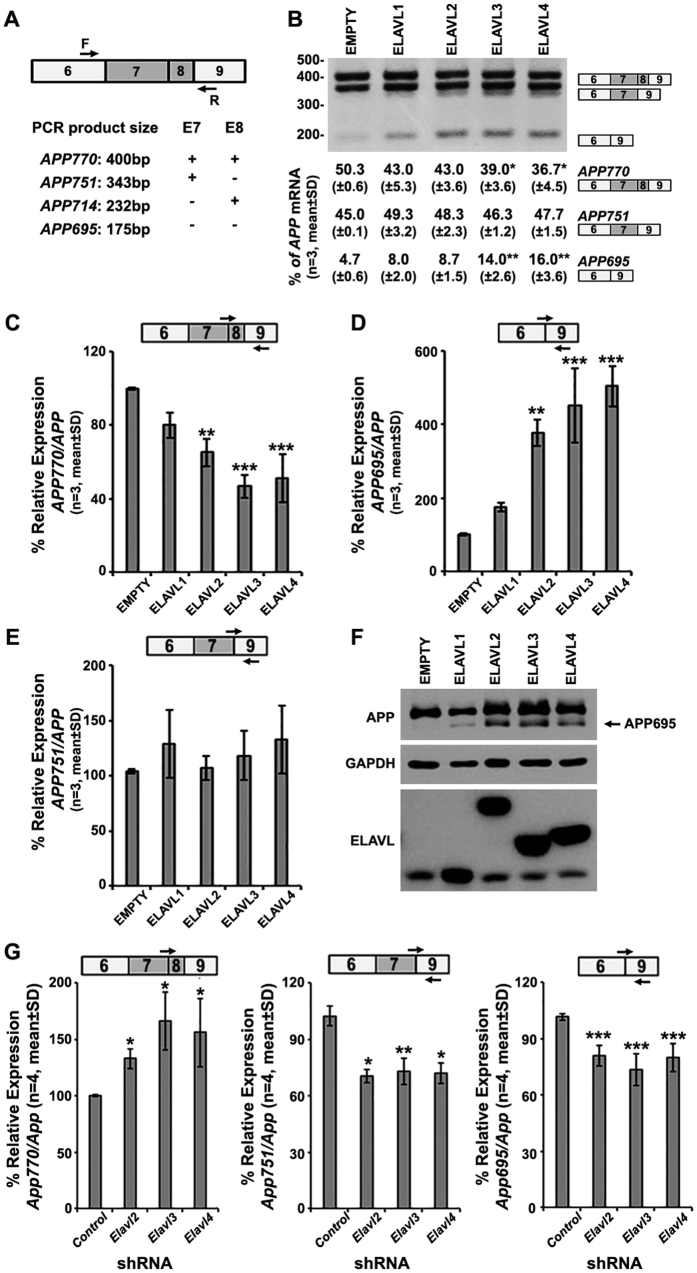
nELAVLs promote the exclusion of exons 7 and 8 from the endogenous APP pre-mRNA. (**A**) Schematic representation of exons 6 to 9 in the APP cDNA and localization of primers used for the simultaneous detection of all transcript variants (*arrows*, F: forward, R: reverse). PCR product size indicative of each transcript is depicted below. (**B**–**F**) Human SK-N-SH cells were transfected with the pCAGGS expression vector bearing either no insert or one ELAVL; the effect of ELAVLs on the inclusion of APP exons 7 and 8 was assayed two days later. (**B**) Splicing pathways were determined by RT-PCR using the primers shown in panel A. Amplification bands resulting from AS of APP exons 7 and/or 8 are indicated. Quantification of the results was performed by scanning densitometry and the percentage (mean ± standard deviation) of each transcript variant is depicted below each lane. (*P < 0.05, **P < 0.01) (**C**–**E**) Total RNA from the transfected SK-N-SH cells was also used in RT-qPCR experiments with primers specific for *APP770* (**C**, *arrows*), *APP695* (**D**, *arrows*) and *APP751* (**F**, *arrows*). Total *APP* cDNA was used for normalization. Bars in graphs correspond to mean ± standard deviation of three independent experiments (**P < 0.01, ***P < 0.001). (**F**) Equal amounts of total protein from lysates of the same transfected SK-N-SH cells were analyzed on SDS-PAGE, immunoblotted with an antibody against APP, ELAVLs and GAPDH, as a loading control. Note that overexpression of ELAVL2, ELAVL3 or ELAVL4 promoted APP695-specific AS. (**G**) Mouse Neuro-2a cells were transfected with an expression vector carrying a shRNA specific for eGFP (control) *Elavl2, Elavl3* or *Elavl4* mRNA. The effect of reduced nELAVL expression on the AS pattern of *App* exons 7 and/or 8 was assayed by RT-qPCR using primers specific for *App770, App751* and *App695*. Total *App* cDNA was used for normalization. Note that Neuro-2a cells with reduced levels of ELAVL2, ELAVL3 or ELAVL4 displayed significant changes in the relative expression of *App770, App751* and *App695*, indicative of suppressed exclusion of exons 7 and 8 from the *App* mRNA. Bars in graphs correspond to mean ± standard deviation of four independent experiments (*P < 0.5, **P < 0.01, ***P < 0.001).

The correlation between nELAVLs and APP695 expression suggests a role for these RBPs in the regulation of APP695-specific AS. To test this hypothesis, SK-N-SH cells, that express no nELAVLs, were transfected with an expression vector bearing either one *Elavl* or no insert (empty) as a control and the effect of ELAVLs on the AS pattern of APP exons 7 and 8 was assayed by RT-PCR with primers that allow the simultaneous detection of all four different splicing events (Fig. 2A). As shown in Fig. 2B, overexpression of ELAVL3 or ELAVL4 led to a significant decrease in the percentage of full-length *APP* mRNA (*APP770*: ANOVA, F_(4,14)_ = 5.429, P < 0.05 and Bonferroni post-hoc) along with a significant increase in the percentage of *APP* mRNA lacking both exons 7 and 8 (*APP695*: ANOVA, F_(4,14)_ = 12.075, P < 0.01 and Bonferroni post-hoc); in contrast, no difference was observed on the percentage of *APP* transcripts lacking either only exon 7 (*APP714*, undetectable) or only exon 8 (*APP751*). Notably, ELAVL overexpression had no effect on the AS pattern of APP exon 15 (see Supplementary Fig. S1A–C). Total RNA from the above transfected cells was also used in a series of RT-qPCR experiments with primers specific for *APP770, APP751* and *APP695*. Our analysis revealed that in contrast to the ubiquitous ELAVL1, overexpression of ELAVL2, ELAVL3 or ELAVL4 resulted in a significant reduction of *APP770* relative expression by 35%, 53% or 49% (Fig. 2C; ANOVA, F_(4,14)_ = 22.573, P < 0.001 and Bonferroni post-hoc), along with a significant 3.8-fold, 4.5-fold or 5-fold upregulation of *APP695*, respectively (Fig. 2D; ANOVA, F_(4,14)_ = 31.707, P < 0.001 and Bonferroni post-hoc). No difference was observed on *APP751* relative expression (Fig. 2E). Induction of APP695-specific AS by nELAVLs was also evident at the protein level (Fig. 2F) and similar results were also obtained from a comparable set of experiments performed in Neuro-2a cells (see Supplementary Fig. S2A–C).

**Figure 3:**
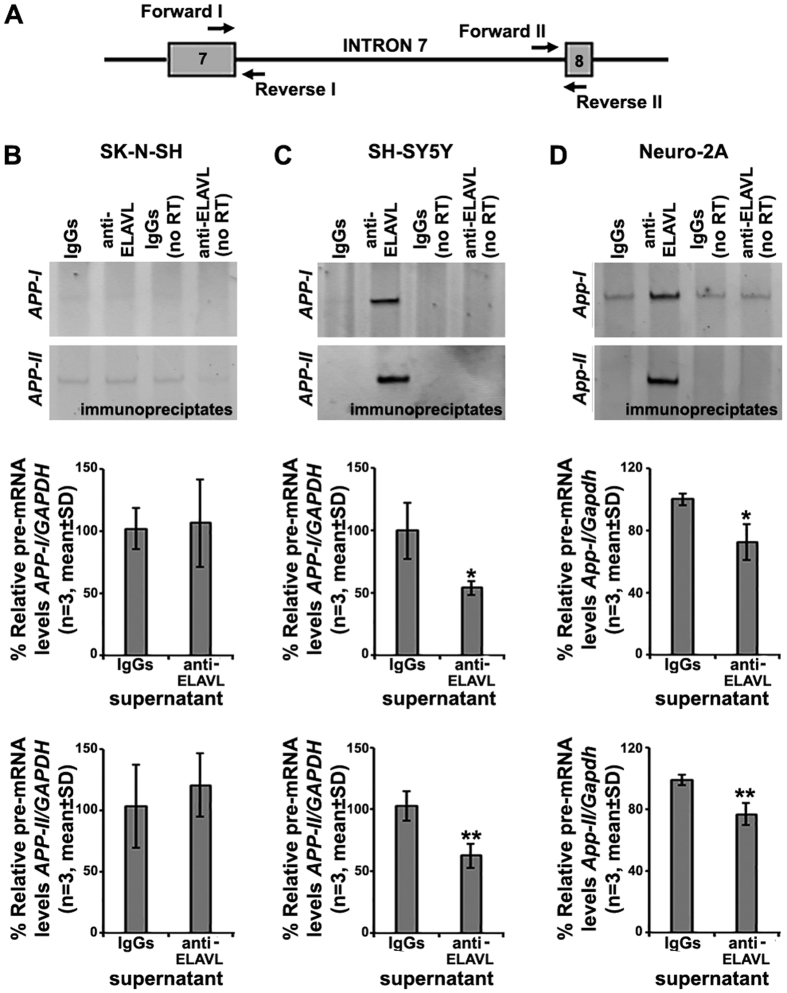
Association of nELAVs with the APP pre-mRNA. (**A**) Localization of primers used for the detection of *APP/App* pre-mRNAs (*arrows*; *APPI/AppI*: Forward I and Reverse I; *APPII/AppII*: Forward II and Reverse II). Boxes indicate exons and bold lines introns. (**B**–**D**) Nuclear extracts prepared from human SK-N-SH (**B**), human SH-SY5Y (**C**) and mouse Neuro-2a (**D**) cells were immunoprecipitated with a mouse anti-ELAVL antibody or mouse IgGs as a control. RNA was isolated from immunoprecipitates, as well as their supernatants and analyzed by semi-quantitative and quantitative RT-PCR, respectively, using specific primers against human or mouse APP pre-mRNA (*arrows*) and intronic GAPDH. Minus RT lanes are included as controls. Note that APP pre-mRNA was detectable in the immunoprecipitate only when lysates from cells expressing nELAVLs were used. Bars in graphs depict mean ± standard deviation of three independent experiments (*P < 0.5, **P < 0.01).

To gain further insight, Neuro-2a cells expressing nELAVLs and APP695, were transfected with an expression vector carrying a shRNA specific for each *nElavl* or GFP mRNA, as a control. Reduction of nELAVL-expression was verified at the mRNA and protein level (see Supplementary Fig. S3) and its effect on the AS pattern of *App* exons 7 and/or 8 was assayed by RT-qPCR. As depicted in Fig. 2G, reduction of ELAVL2, ELAVL3 or ELAVL4 levels resulted in increased inclusion of *App* exons 7 and 8 by 33%, 66% and 56%, respectively (*App770/App*: ANOVA, F_(3,15)_ = 8.118, P = 0.003 and Bonferroni post-hoc). Consistently, a significant decrease in the relative expression of *App751* by ~30% (Fig. 3C, ANOVA, F_(3,15)_ = 32.182, P < 0.001 and Bonferroni post-hoc) and of *App695* by ~20% (Fig. 3D, ANOVA, F_(3,15)_ = 14.386, P < 0.001 and Bonferroni post-hoc) was also observed. Collectively, the experiments described above demonstrate that nELAVLs play a critical role in the exclusion of exons 7 and 8 from both the human and the mouse APP mRNA.

### nELAVLs interact with the APP pre-mRNA

We next examined whether nELAVLs interact with the *APP* pre-mRNA by performing RIP using a mouse anti-ELAVL antibody, followed by RT-PCR with primers amplifying sequences flanking exon/intron boundaries of exons 7 and 8 (Fig. 3A). To discriminate between ELAVL1- and nELAVL-binding, ELAVL complexes were immunoprecipitated from nuclear extracts of SK-N-SH cells expressing only *ELAVL1* (Fig. 3B) and SH-SY5Y cells expressing all *ELAVLs* (Fig. 3C). As shown in Fig. 3, *APP* pre-mRNA was detected only in the immunoprecipitate of SH-SY5Y cells, whereas in both immunoprecipitates no *GAPDH* pre-mRNA was present (data not shown). To verify this selective association, *APP* pre-mRNA levels were also assayed in the ELAVL-immunoprecipitates’ supernatant by RT-qPCR; consistently, compared to IgGs controls, *APP* pre-mRNA appeared depleted in the supernatant only when nuclear extracts from SH-SY5Y cells were used (Fig. 3C; Student’s *t*-*test*, *P < 0.05, **P < 0.01). Finally, significant levels of *App* pre-mRNA were also detected in ELAVL-complexes immunoprecipitated from nuclear extracts of Neuro-2a cells (Fig. 3D, Student’s *t*-*test*, *P < 0.05, **P < 0.01). Our results combined point to a preferential association of nELAVLs to the human and mouse APP pre-mRNA, supporting their role in neuron-specific processing of these pre-mRNAs.

### nELAVLs suppress the inclusion of either both exons 7 and 8, or only exon 8 in transcripts from artificial minigenes

**Figure 4:**
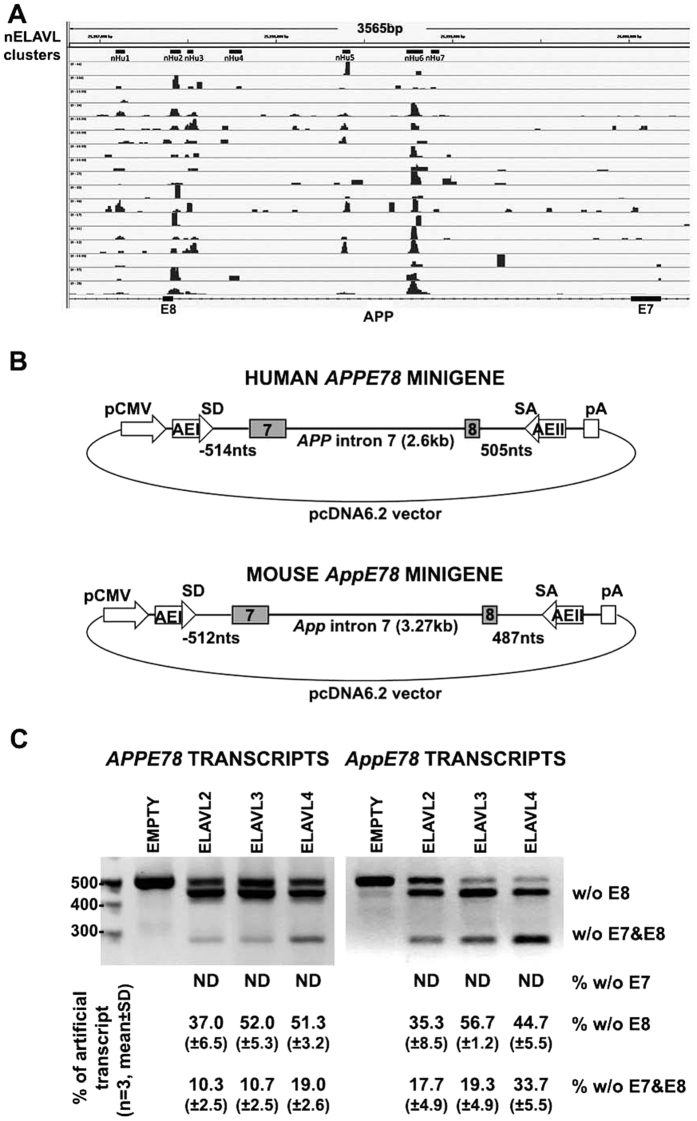
nELAVLs promote APP exon 7 and/or 8 skipping from APPE78 minigene transcripts. (**A**) Normalized nELAVL binding map in human *APPE78* region. IGV Browser snapshot depicting the nELAVL binding profiles in all 17 human brain samples surrounding exons 7 and 8. Note that there is a cluster of nELAVL binding sites spanning the intronic region in-between exons 7 and 8, while three of the identified peaks are found in close proximity to exon 8. nELAVL peaks, are located on chromosome 21: 25,996,827–26,000,394 bp. (**B**) Schematic representation of human *APPE78* and mouse *AppE78* minigenes. Boxes indicate exons (AE: artificial exon; pA: poly-A signal; pCMV: CMV promoter; SA: splice acceptor, SD: splice donor). (**C**) Human SK-N-SH cells were co-transfected with the human or the mouse APPE78 minigene along with the pCAGGS expression vector bearing either no insert (empty) or one of the three nELAVLs. Splicing pathways were determined by RT-PCR using primers specific for artificial exons I and II. Amplification bands resulting from AS of APP exons 7 and/or 8 are indicated. Quantification of the results was performed by scanning densitometry and the percentage (mean ± standard deviation) of each transcript variant is depicted below each lane. Note that apart from transcripts lacking both APP exons, significant levels of transcripts lacking only exon 8 were also observed upon nELAVL-overexpression.

It has been predicted that nELAVLs regulate AS of exons flanked by U-rich sequences interspersed with purine residues, usually within 250 nucleotides (nts) of intron/exon boundaries^26^. Our initial analysis of human and mouse genomic regions surrounding APP exons 7 and 8 revealed several sequences encoding for such U-rich blocks close to splice sites, as well as deep in the intervening intron 7 (see Supplementary Tables S2 and S3). To confirm that these are nELAVL-binding sites, we re-analyzed 17 human brain HITS-CLIP datasets for nELAVL-RNP complexes (accession number GSE53699)^32^ and show a large number of CLIP tags in these predicted U-rich blocks (Fig. 4A). To validate that these blocks are the *cis*-acting elements responsible for nELAVL-induced exon 7 and 8 skipping, we initially constructed two artificial minigenes containing either the human (*APPE78*) or the mouse (*AppE78*) genomic sequence, extending from ~500 nts upstream of exon 7 to ~500 nts downstream of exon 8 (Fig. 4B). Each minigene was then co-transfected along with an ELAVL2-, ELAVL3- or ELAVL4- expression vector in SK-N-SH cells and the effect of nELAVLs on the inclusion of exons 7 and 8 was assayed by RT-PCR using primers specific for the flanking artificial exons (Fig. 4B). As shown in Fig. 4C, all nELAVLs -but most efficiently ELAVL4, which for that reason was used in all our subsequent experiments- promoted the generation of transcripts lacking exons 7 and 8. In general, nELAVLs displayed a more dramatic effect on the AS of mouse transcript compared to the human, probably due to other differences in the *cis*-acting elements among the two species. Interestingly, all nELAVLs appeared very potent in inducing exon 8 –but not exon 7- skipping separately, a finding that was not observed in our initial transfection experiments. It thus seems that despite differences in the regulation of exon 8 AS between endogenous and *APPE78/AppE78* minigenes, the latter constructs appear sufficient for evaluating the regulation of APP695-specific AS by nELAVLs.

### Identification of minimal sequences required for nELAVL-mediated exclusion of APP exon 8

**Figure 5:**
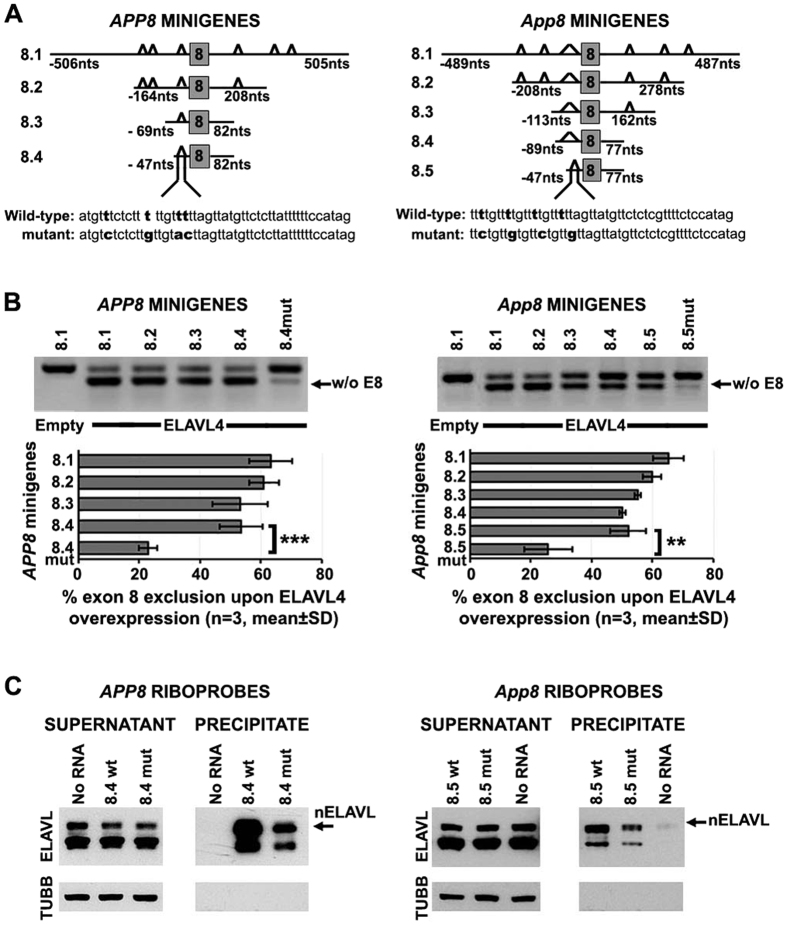
Identification of a U-rich element important for nELAVL-mediated APP exon 8 skipping. (**A**,**B**) Schematic diagrams depicting the regions of human (**A**) and mouse (**B**) APP locus used for the generation of *APPE8* and *AppE8* minigenes, respectively. The gray box represents exon 8, bold lines its flanking intronic sequences, whose length is depicted, and triangles segments encoding for U-rich regions in the pre-mRNA. The 47 nt upstream sequence of wild-type and mutant *APPE8.4* and *AppE8.5* is also shown. (**C**,**D**) Human SK-N-SH cells were co-transfected with human (**C**) or mouse APPE8 (**D**) minigenes and the pCAGGS expression vector bearing no insert (empty) or ELAVL4. Splicing pathways were determined by RT-PCR using primers specific for the artificial exons of the minigene vector. Amplification bands resulting from exon 8 skipping are indicated. Quantification of the results was performed by scanning densitometry and the percentage of exon 8 exclusion (mean ± standard deviation) is displayed in graphs (**P < 0.01, ***P < 0.001). Note that ELAVL4 efficiently promoted exclusion of exon 8 from all wild-type artificial transcripts, but this effect was compromised in the case of mutant ones. (**E**,**F**) Biotinylated RNA segments transcribed from wild-type and mutant *APPE8.4* (**E**) and *AppE8.5* (**F**) minigenes were tested for nELAVL-binding after incubation with lysates of Neuro-2a cells and pull-down using streptavidin beads. The presence of nELAVLs was assayed by immunoblotting. Note that nELAVLs strongly associated with the wild-type transcripts, but this association was weaker when the mutant transcripts were used.

Since nELAVLs appeared very potent in inducing APP exon 8 skipping from the generated minigenes, we initially examined the *cis*-elements that affect this process. For this reason, we generated a series of minigenes consisting of the human (*APPE8*) or mouse (*AppE8*) exon 8 and progressively shorter regions of their flanking introns fostering a progressively reduced number of U-rich blocks (Fig. 5A). Each minigene was co-transfected into SK-N-SH cells along with an ELAVL4-expressing vector (or the empty vector as a control) and the effect of ELAVL4 on exon 8 inclusion was assayed by RT-PCR. Overall, co-transfection of each minigene with *Elavl4* resulted in the generation of two transcripts and in all cases 50–60% of generated transcripts did not contain exon 8; no such effect was evident in control co-transfections with the empty expressing vector (Fig. 5B). Notably, *APPE8.4* and *AppE8.5* primary transcripts contained only one U-rich block spanning the last 47 nts of intron 7 that are highly conserved in human, mouse and rat (Fig. 5A and data not shown). To test the importance of this U-rich region in ELAVL4-mediated exon 8 exclusion, point mutations were inserted into *APPE8.4* and *AppE8.5* (Fig. 5A) and wild-type and mutant minigenes were co-transfected in SK-N-SH cells along with *Elavl4* (Fig. 5B). Our analysis showed that compared to wild-type transcripts that were processed to exclude exon 8 at 54% (*APPE8.4*) and 52% (*AppE8.5*), mutant *APPE8.4* and *AppE8.5* transcripts showed significantly reduced exon 8 exclusion at 23% (Student’s *t*-*test*, ***P < 0.001) and 26% (Student’s *t*-*test*, ***P < 0.001), respectively. To verify that these sequences are nELAVL-targets, biotinylated riboprobes generated by wild-type and mutant *APPE8.4/AppE8.5* minigenes were incubated with Neuro-2a whole cell lysates and the association of nELAVLs was assayed by immunoblotting after pull-down with streptavidin-coated beads. As shown in Fig. 5C, nELAVLs strongly associated with wild-type transcripts, but this association was reduced in the case of mutants. Notably, ELAVL1-binding was also evident, but with a much weaker affinity. Taken together, our results indicate that nELAVLs induce exon 8 skipping from both the human and mouse APP pre-mRNA through binding to a conserved U-rich sequence immediately upstream of this alternative exon; all other U-rich sequences flanking this exon play a minor, if any role, in this process.

### Concurrent binding of ELAVL4 to sequences located upstream and downstream of APP exon 7 is required for its skipping

**Figure 6:**
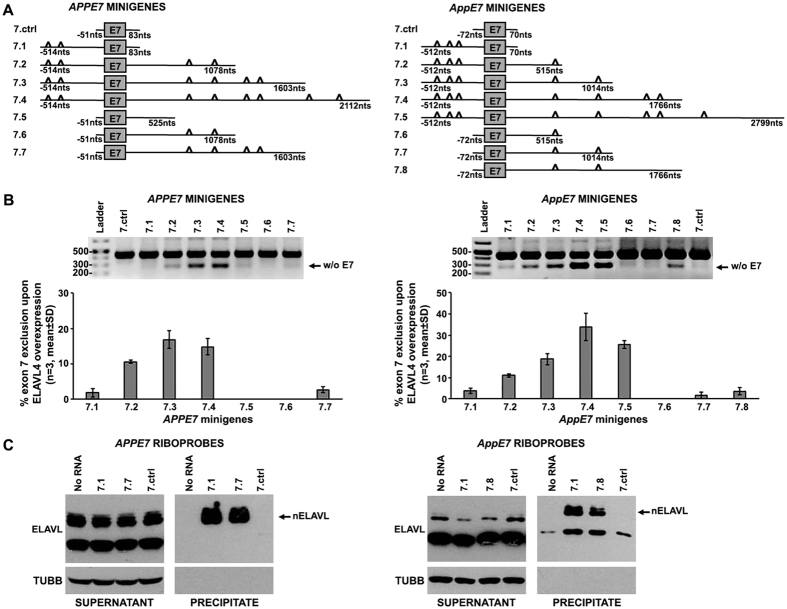
nELAVL-binding to sequences located both upstream and downstream of exon 7 is required for nELAVL-mediated APP exon 7 exclusion. (**A**,**B**) Schematic representation of human (**A**) and mouse (**B**) APP genomic regions used for the generation of *APPE7* and *AppE7* minigenes, respectively. The gray box corresponds to exon 7, bold lines to its flanking intronic sequences, whose length is indicated and triangles to segments encoding for U-rich sequences in the pre-mRNA. (**C**,**D**) Human SK-N-SH cells were co-transfected with either *APPE7* (**C**) or *AppE7* (**D**) minigenes and the pCAGGS expression vector bearing ELAVL4. Splicing pathways were determined by RT-PCR using primers specific for the artificial exons of the minigene vector. Amplification bands of transcripts lacking exon 7 are shown. Quantification of the results was performed by scanning densitometry. Bars in graphs depict the percentage of exon 7 skipping (mean ± standard deviation). Note, that ELAVL4 promoted exon 7 exclusion only from transcripts containing U-rich sequences both upstream and downstream of this exon (**E**,**F**) Biotinylated RNA probes transcribed from the indicated human (**E**) and mouse (**F**) APPE7 minigenes were tested for nELAVL-binding after incubation with lysates of Neuro-2a cells and pull-down using streptavidin beads. The presence of nELAVLs was assayed by immunoblotting. Note, that nELAVLs strongly associated with both flanking intronic regions of APP exon 7.

Based on our above observations, to identify the *cis*-elements on APP pre-mRNA that participate in nELAVL-mediated exclusion of exon 7, we initially generated a human (*APPE7.1*) and a mouse (*AppE7.1*) minigene encoding for transcripts containing U-rich elements close to the upstream splice site of exon 7 (Fig. 6A). Interestingly, similar to control minigenes lacking T-rich intronic regions, co-transfection of *APP7.1* or *App7.1* with *Elavl4* resulted in the generation of only full-length transcripts (Fig. 6B), indicating that binding of ELAVL4 upstream of exon 7 is not sufficient for its skipping. Since our HITS-CLIP analysis revealed strong nELAVL-binding to U-rich blocks located deep in intron 7 (Fig. 4A), we next examined whether these sequences participate in nELAVL-mediated APP exon 7 skipping. Therefore, we generated a series of human (*APPE7.2*-*4*) and mouse (*AppE7.2*-*7.5*) minigenes consisting of the same upstream intronic region, exon 7 and varying lengths of the downstream intron encoding for progressively more U-rich blocks (Fig. 6A). Co-transfection of *Elavl4* with each of the latter minigenes promoted the generation of transcripts lacking exons 7; notably, transcripts generated from minigenes with progressively more nELAVL-binding sites downstream of exon 7, were processed to exclude exon 7 at progressively higher levels (Fig. 6B). Nevertheless, these downstream sequences were not sufficient for ELAVL4-mediated exclusion of exon 7, in the absence of U-rich blocks in the upstream intron 6 (*APPE7.5*-*7* and *AppE7.6*-*8*; Fig. 6B). Binding of nELAVLs to sequences both upstream and downstream of exon 7 was verified by RNA pull-down assays using biotinylated riboprobes from human and mouse minigenes containing T-rich regions either only upstream (*APP7.1* or *AppE7.1*), or only downstream (*APP7.7* and *AppE7.8*) of exon 7 (Fig. 6C). Taken together, our results indicate that concurrent binding of nELAVLs both upstream and deeply downstream of exon 7 is required for its exclusion from the human and mouse APP mRNA.

### ELAVL4 interfere with the recognition of 3′ splice site of APP exon 7 and exon 8 by reducing U2AF65 binding

**Figure 7:**
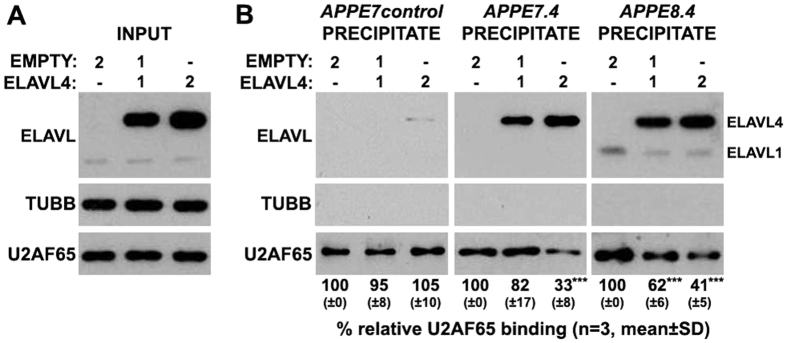
ELAVL4 protein interferes with the binding of essential splicing factor U2AF65 upstream of APP exons 7 and 8. Human SK-N-SH cells were co-transfected with two expression plasmids carrying either no insert (empty) or ELAVL4 in ratios depicted above each lane. (**A**) In order to ensure the presence of different ELAVL4 protein levels among the three conditions, equal amounts of total protein from lysates of the transfected SK-N-SH cells (input) were analyzed on SDS-PAGE and immunoblotted with antibodies against ELAVLs and β-TUBULIN (TUBB) as a loading control; membranes were also probed against U2AF65, to verify that ELAVL4 overexpression does not alter U2AF65 expression. (**B**) Whole cell lysates from the same transfected cells were used in a series of pull down assay using streptavidin beads and biotinylated RNA probes transcribed from the indicated human minigenes. The presence of U2AF65 and ELAVLs in the precipitates was assayed by immunoblotting. Quantification of the results was performed by scanning densitometry and the percentage (mean ± standard deviation) change of U2AF65-binding upon ELAVL4 overexpression is shown below each lane. Note, that ELAVL4-binding to these transcripts resulted in a reduction of U2AF65 binding (***P < 0.001).

Given that ELAVL4-binding upstream of APP exons 7 or 8 is necessary for their skipping, we next examine whether ELAVL4 hinders the interaction of the essential splicing factor U2AF65 (U2 small nuclear ribonucleoprotein (snRNP) auxiliary factor 65 kDa) with the upstream 3′ splice site of these exons. For this reason, SK-N-SH cells were co-transfected with progressively increased amounts of an ELAVL4-expression plasmid and whole cell lysates of transfected cells were used in a series of RNA pull down assays using the biotinylated riboprobes *APPE7.4* and *APPE8.4*, to which nELAVLs bind and efficiently induce skipping of exons 7 and 8, respectively; *APPE7control* bearing no nELAVL-binding sites, was also used as control. As shown in Fig. 7, ELAVL4 reduced U2AF65 binding to *APPE7.4* and *APPE8.4* transcripts in a dose-dependent way (*APPE7.4*: ANOVA, F_(2,8)_ = 31.337, P = 0.001 and Bonferroni post-hoc; *APPE8.4*: ANOVA, F_(2,8)_ = 119.805, P < 0.001 and Bonferroni post-hoc) and this effect was not observed when control transcripts were used. Notably, ELAVL4 appeared to be more potent in displacing U2AF65 from *APPE8.4* transcript, as a ~40% reduction was also observed in the presence of half the amount of ELAVL4. It thus appears that nELAVLs may interfere with the binding of U2AF65 to the 3′ splice site of APP exons 7 and 8.

### AUF-1 cooperates with nELAVLs in the regulation of APP695-specific AS

**Figure 8:**
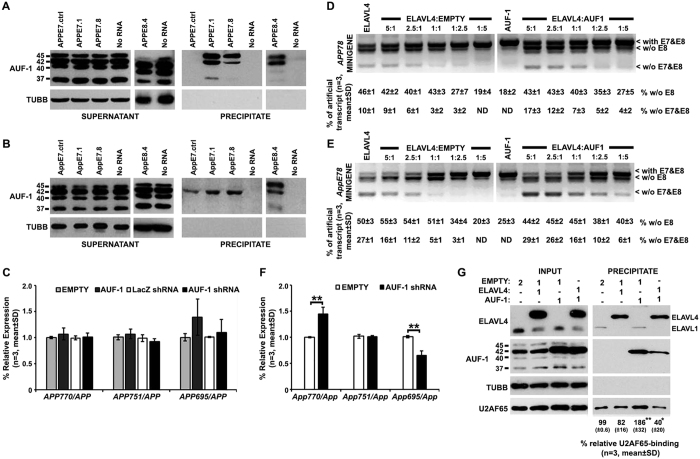
AUF-1 facilitates ELAVL4-mediated APP695-specific AS. Biotinylated RNA probes transcribed from human (**A**) and mouse (**B**) APP minigenes were tested for AUF-1 binding. Note, that AUF-1 isoforms p42 and/or p45 bind to all transcripts shown to interact with nELAVLs. (**C**) SK-N-SH cells were transfected with the DNA3.1 expression vector bearing either no insert (empty) or p42 AUF-1, or with the pENTR/U6 vector carrying a shRNA specific for LacZ or all *AUF*-*1* mRNAs. Inclusion of APP exons 7 and 8 was assayed by RT-qPCR with primers specific for *APP770, APP695* and *APP751*. Total *APP* cDNA was used for normalization. Bars in graphs correspond to mean ± standard deviation of three independent experiments. (**D**,**E**) SK-N-SH cells were co-transfected with the human (**D**) or the mouse (**E**) APPE78 minigene along with two expression vectors, one bearing ELAVL4 and the other one carrying no insert (empty) or p42 AUF-1, in ratios depicted. Splicing pathways were determined by RT-PCR, as described in Fig. 5. Quantification of the results was performed by scanning densitometry. Note, that AUF-1 enhances ELAVL4-mediated exclusion of APP exons 7 and 8. (**F**) Neuro2a cells were transfected with the pSilencer vector bearing either no insert (empty) or a shRNA targeting all *Auf*-*1* transcripts. Inclusion of APP exons 7 and 8 was assayed by RT-qPCR, as described above (**P < 0.01). Note, that in the presence of nELAVLs, reduction of AUF-1 levels results in reduced APP695-specific AS. (**G**) SK-N-SH cells were co-transfected with two out of three expression plasmids carrying no insert (empty), ELAVL4 or p42 AUF-1 in ratios depicted. Whole cell lysates from these cells were used in a series of pull down assay using streptavidin beads and APP7.4 biotinylated riboprobe. The presence of ELAVLs, AUF-1 and U2AF65 in precipitates was assayed by immunoblotting. Quantification of the results was performed by scanning densitometry and the percentage (mean ± standard deviation) change of U2AF65-binding is shown below each lane. Note that concurrent binding of both proteins in the transcript resulted in a significant reduction of U2AF65 binding. (*P < 0.05, **P < 0.01).

nELAVLs have been shown to regulate AS through binding to intronic sequences in competition with other splicing factors^27^,^29^. Given that AUF-1 and TIA-1 have been shown to share common targets with ELAVLs^27^,^28^,^33^,^34^, we initially examined whether regions of *APP* pre-mRNA essential for nELAVL-induced exon 7 and 8 skipping are also targets of these RBPs. Our analysis revealed a strong association of AUF-1 (especially isoforms p45 and p42)- but not TIA-1- with riboprobes from human or mouse APP pre-mRNA shown to be also bound by nELAVLs (Fig. 8A,B and data not shown). To test the functional importance of this association SK-N-SH cells were transfected with either *AUF*-*1* (encoding for p42 isoform) or an shRNA targeting all *AUF*-*1* transcripts. Intriguingly, changes in AUF-1 level (see Supplementary Fig. S4A) had no effect on the AS pattern of *APP* exons 7 and 8, as assessed by RT-qPCR (Fig. 8C). To examine whether nELAVLs compete with AUF-1 to regulate APP695-specific AS, a series of co-transfection experiments was performed using the human or mouse APPE78 minigene and a combination of ELAVL4- and p42 AUF1- expression plasmids in various ratios (Fig. 8D,E). Consistent with the role of ELAVL4 as a splicing factor, in our control experiments, levels of alternative transcripts were gradually reduced following the decrease in the amount of ELAVL4-expression plasmid. Interestingly, when AUF-1 was co-expressed with ELAVL4, levels of transcripts lacking both exons 7 and 8, but not those lacking only exon 8, were significantly increased by 2-3 folds compared to their controls, suggesting that AUF-1 enhances ELAVL4-induced APP695 specific AS. To verify the latter synergistic function, *Auf*-*1* expression was silenced in Neuro2a cells that express significant levels of nELAVLs and APP695 (see Supplementary Fig. S4B). As predicted, reduction of AUF-1 levels in these cells resulted in a significant 45% increase of *App770* along with a significant 35% decrease of *App695* relative expression (Student’s *t*-*test*, P = 0.002 and P = 0.004, respectively); in contrast no difference was observed on *App751* abundance. Finally, to test whether AUF-1 facilitates U2AF65 displacement by nELAVLs, SK-N-SH were co-transfected with equal amounts of ELAVL4- and AUF-1- expression plasmids and whole cell lysates of the transfected cells were used in RNA pull down assays using *APPE7.4* biotinylated riboprobes. As shown in Fig. 8G, compared to controls, in the presence of suboptimal levels of ELAVL4, no significant reduction of U2AF65 binding to APP7.4 transcript was observed. Interestingly, upon AUF-1 overexpression U2AF65-binding was increased by ~90%; nevertheless when ELAVL4 was co-expressed along with AUF-1 a significant decrease of U2AF65-binding was observed compared to all other conditions (ANOVA, F^3^,^11^ = 25,391, P < 0.001 and Bonferroni post-hoc). Taken together, our observations suggest that ELAVL4 uses AUF-1 as a cofactor to promote exclusion of exons 7 and 8 from the *APP* pre-mRNA.

## Discussion

In the present study, we report that nELAVLs, as opposed to ubiquitous ELAVL1, are critically involved in neuronal-specific AS of human and mouse APP pre-mRNA, by promoting the exclusion of exons 7 and 8. Moreover, we demonstrate the mechanism by which nELAVLs regulate this process and provide evidence supporting that AUF-1 facilitates nELAVL-induced APP695-specific AS.

By using a series of minigenes, we identified *cis*-elements on the APP pre-mRNA important for nELAVL-mediated exon 7 or 8 skipping and report that an evolutionary conserved U-rich sequence immediately upstream of exon 8 acts as a splicing repressor and is important for nELAVL-mediated skipping of this exon. Our observations are consistent with the position-dependent model for AS regulation by RBPs, according to which preferential binding of RBPs in close proximity to the 3′ splice site of the target exon is usually associated with exon skipping, while binding within approximately 300 nts downstream of the exon is associated with exon inclusion^35^,^36^,^37^. However, this simple position-based rule did not apply to nELAVL-mediated APP exon 7 skipping, that appeared to be dependent on the concurrent binding of nELAVLs to U-rich sequences both upstream and downstream of this target exon. Our latter result is in agreement with the nELAVL-RNA map recently reported^26^; consistently nELAVLs have been shown to promote exon 23a exclusion from neurofibromin 1 (*NF1*) pre-mRNA by binding on either flanking intron of this exon^29^. All previous studies examining the role of nELAVLs in AS have reported nELAVL-binding in close proximity to splice sites^27^,^29^,^30^. Herein, we provide evidence that nELAVLs are also able to control AS by binding to distal intronic sequences, as ELAVL4-induced exon 7 exclusion was dependent on the interaction of ELAVL4 with U-rich sequences located deeply in the downstream intron 7.

Notably, although nELAVLs were able to promote exclusion of either exon 7 or exon 8 from their respective artificial transcripts, when their effect on the endogenous APP pre-mRNA was examined, a significant change was observed only in the level of transcripts lacking both these two exons. It thus appears that in the case of nELAVL-mediated regulation of APP AS, adjacent exons 7 and 8 behave as a “joint cassette exon”. An interesting question arises how nELAVLs promote the concurrent exclusion of these two exons from the endogenous mRNA. Given the existence of nELAVL-binding sites upstream of exon 7, as well as throughout its downstream intron 7, a possible mechanism in which nELAVLs regulate splicing is by binding and multimerizing along the pre-mRNA. Along the same lines, herein we show that ELAVL4-mediated exon 7 skipping was progressively more efficient on transcripts with progressively more distal U-rich blocks. Consistently, nELAVLs, as well as the *Drosophila* homologue ELAV exists as multimers and multimerized ELAV has been shown to be functionally important on *Drosophila* ewg RNA AS^24^,^38^,^39^. Multimerization of nELAVLs may provide a mechanism to bring into close proximity the distant RNA-processing signals of APP exons 6 and 9 by looping out the intronic sequence containing exons 7 and 8 and thus increasing the frequency of their skipping. Alternatively, it might interfere with the interaction of spliceosomal components and thus affect proper exon recognition. Both the above mechanisms have been proposed for the splicing regulator hnRNPA1^40^,^41^ and nELAVLs have been shown to promote *NF1* exon 23a skipping by preventing binding of core splicing factors^29^. Herein, we show that nELAVLs interfere with binding of U2AF65 to the 3′ splice site of both exons 7 and 8. Interestingly, although in the case of exon 8 it appears to involve competition for binding to the same target, this is unlikely to account for the reduced binding of U2AF64 upstream of exon 7, given that nELAVLs bind to sequences located more than 300 nts away from intron/exon boundaries of exon 7.

In all experiments described herein, although nELAVLs promoted exclusion of APP exons 7 and 8, abundant expression of transcripts containing these two exons was still observed. We speculated that this might be attributed to either the presence of endogenous factors that may function competitively, or to the deficiency of an unknown splicing regulator in the transfected cells. It has been previously reported that nELAVLs regulate neuron-specific AS of the *calcitonin/GCRP* and *NF1* pre-mRNAs, by binding to intronic U-rich sequences in competition with the ubiquitously expressed TIA-1/TIAR, which will otherwise promote exon inclusion^27^,^29^. A similar mechanism is unlikely to apply in the case of nELAVL-regulated APP AS, since TIA-1 did not interact with the APP pre-mRNA regions participating in this process. Recently, AUF-1 has been shown to share many common target RNAs with ELAVL1 and compete or cooperate with ELAVL1 in the translational activation of these targets^33^. Moreover, AUF-1 binding has been observed at intronic sites and silencing of *AUF*-*1* has been associated with alterations in the levels of selected groups of alternative transcripts^34^. In the present study, we provide evidence that AUF-1 acts as a cofactor of nELAVLs in the regulation of APP695-spesific AS; AUF-1, more specifically isoforms p45 and/or p42, binds to APP pre-mRNA regions shown to be nELAVL-targets and despite its minor effect on the AS pattern of exons 7 and 8 in the absence of nELAVLs, AUF-1 silencing in cells expressing nELAVLs results in increased inclusion of exon 7 and 8 in APP transcripts. To our knowledge, this study provides the first evidence that AUF-1 directly modulates AS events. Although the mechanism, by which AUF-1 facilitates nELAVL-induced APP695-specific awaits further investigation, an interesting possibility is that local changes in RNA structure may account for this synergistic effect. Previous studies have demonstrated that binding of AUF-1 is able to alter the local conformation of its RNA targets^42^,^43^. Although the functional significance of local RNA structural remodeling by AUF-1 remains unclear, a logical functional consequence of this effect is to control accessibility for additional RNA-binding factors and thus influence RNA fate depending on the presence of RBPs within the cell. In that respect, it’s noteworthy that in the absence of nELAVLs binding of p42 AUF-1 to APPE7 transcripts increased U2AF65-binding, whereas in the presence of ELAVL4 enhanced nELAVL-mediated U2AF65 displacement, probably through facilitating ELAVL4-binding to this target sequence.

Taken together our results suggest a neuronal mechanism, conserved in human and mouse, for the regulation of APP695 production by nELAVLs. To date a few factors have been proposed to regulate APP exon 7 and/or exon 8 skipping. RNA-binding proteins Fox -1 and -2 (RBFOX1 and 2), have been shown to induce exon 7, but not exon 8, exclusion^44^, whereas the Elav-like family 1 (CELF1), a CUG-binding protein, has been shown to induce *APP* exon 8 skipping^45^. Intriguingly, APP714 lacking only exon 7 has a relative high expression in skeletal muscles^6^ where RBFox1 and 2 are abundantly expressed^46^, whereas APP751 lacking only exon 8 and CELF1 are ubiquitously expressed^3^,^6^,^47^. Interestingly, SC35 and hnRNPA1 have been also involved in the exclusion of both *APP* exons 7 and 8^13^. However, neither SC35, nor hnRNPA1 are neuronal-specific^48^,^49^. APP695, lacking both exons 7 and 8, is predominantly expressed in neurons^3^,^6^, where nELAVLs are also predominantly expressed^20^,^26^. Herein, we provide evidence that a similar association exists also in cell lines *in vitro* and manipulation of nELAVL levels in neuroblastoma cells leads to changes of endogenous APP695 relative expression. Although keeping in mind that neuroblastoma cells have been shown to express alterations in AS patterns compared to neuronal cells^50^, our bioinformatics’ analysis showing a strong correlation between *APP695* and *nELAVLs* levels in the human brain, suggests that nELAVLs may play an orchestrating role in the regulation of APP695-specific RNA processing *in vivo*.

APP695 is principally neuronal and in neurons is present at relatively high levels compared to the other two major isoforms APP770 and APP751^3^,^6^. Until recently no conclusive functional differences have been ascribed to these different isoforms apart from the protease inhibitor role of the Kunitz-type protease inhibitor (KPI) domain encoded by exon 7; nevertheless, efficiency of Aβ production appears to be affected by the APP isoform^13^,^14^,^51^ and alterations in neuronal splicing of *APP* have been observed in the brain of AD patients, with numerous studies reporting a decrease in neuronal *APP695* along with an increase in full-length *APP770*^9^,^10^,^11^,^12^. Of note, unlike latter reports, herein we failed to detect differences in *APP* isoform levels (as well as *nELAVL* levels) between control and AD samples, most possibly due to the small sample size and the observed high intra-group heterogeneity (see also original article 32). However, taking into account our results suggesting a strong correlation of *APP695* and *nELAVL* levels in the human brain, we speculate that a deficiency of nELAVLs could contribute to the reduced *APP695* relative levels reported elsewhere^9^,^10^,^11^,^12^. Although this speculation awaits future investigation, it’s noteworthy that previous studies have reported alterations in nELAVL protein levels^31^,^52^ or binding profile^32^ in the brain of AD patients.

Taken together, our results suggest that nELAVLs play an orchestrating role in the regulation of neuronal APP695-specific RNA processing, facilitated by AUF-1. Since nELAVLs have been also involved in the regulation of APP steady-state levels^31^, it appears that they play a key role in the post-transcriptional regulation of APP expression, alterations of which have been linked to AD.

## Methods

### Plasmids

To generate cDNA sequences of *Elavl1, Elavl2, Elavl3*, and *Elavl4*, reverse transcription (RT) and polymerase chain reaction (PCR) were carried out using RNA isolated from CD1 mouse cells brain and specific primers for each cDNA (see Supplementary Table S5). PCR products were inserted into the pcDNA3.1/V5-His-TOPO vector (Invitrogen), and the cDNAs were further subcloned into the EcoRV and XhoI sites of pCAGGS-IRES-GFP vector. In order to generate shRNA specific for human *AUF*-*1* and LacZ mRNA, Block-iT U6 RNAi Entry vector Kit (Invitrogen) as well as *AUF*-*1* shRNA oligos (see Supplementary Table S5) and LacZ shRNA oligos (provided by Invitrogen) were used. The AUF-1 expression plasmids^53^ were a kind gift of Dr. P. Naveilhan (University of Nantes, France), the five shRNA expression plasmids, each containing a shRNA targeting *Elavl2, Elavl3* and *Elavl4* or eGFP transcript^30^ were kindly provided by Dr. H. Lou (Case Western Reserve University, Cleveland, USA) and the two pSilencer plasmids containing no insert or a shRNA targeting all *Auf*-*1* transcripts^33^, were provided by Dr. M. Gorospe (National Institutes of Health, Baltimore MD, USA).

The minigene vector constructed consists of sequences from the pcDNA6.2-GW/EmGFP plasmid (backbone vector; Invitrogen), the splice donor sequence of EF1 exon1 [SD, a gift of Dr. A. Klinakis, Biomedical Research Foundation Academy of Athens (BRFAA), Greece] and an adenoviral splice acceptor (SA, provided by Dr. L. Zagoraiou, BRFAA, Greece). SD and SA sequences were PCR-amplified using SD and SA primers, respectively (see Supplementary Table S5). SD product was cloned into the pcDNA3.1/V5-His-TOPO vector, digested with BamHI and XhoI, blunted and inserted into the DraI site of pcDNA6.2-GW/EmGFP vector replacing the EmGFP coding sequence. SA product was digested with BamHI and XhoI and inserted into the same sites of the pcDNA6.2 vector.

The APPE78 sequences used in this study consist of a part of the human or mouse APP genomic sequence extending from ~500 nucleotides (nts) upstream of exon 7 up to ~500 nts downstream of exon 8. To generate *APPE78* and *AppE78* minigenes, human and mouse sequences were PCR-amplified from HEK293 cell and mouse CD1 genomic DNA, using APP7_514 F and APP8_505 R or App7_512 F and App8_487 R primers, respectively. PCR products were digested with NotI and SalI and cloned into the same sites of the above minigene vector. To obtain the series of APPE7 (containing exon 7 and parts of its flanking introns) and APPE8 (containing exon 8 and parts of its flanking introns) minigenes, desired sequences were PCR-amplified using as templates *APPE78* or *AppE78* sequences and the appropriate combination of primers (primer sequences are summarized in Supplementary Table S5). PCR products were digested with NotI and SalI and were inserted either into the same sites of the minigene vector or into the NotI and XhoI sites of the DNA3.1 vector that was further linearized and used as a template for *in vitro* transcription.

### Cell culture and transfections

Human HEK293, HeLa, SK-N-SH, SH-SY5Y and mouse Neuro-2a cells were cultured in Dulbecco’s modified Eagle’s medium (DMEM) supplemented with 10% fetal bovine serum (FBS) (Invitrogen). Mouse CAD cells were maintained in DMEM containing 8% FBS and mouse primary cortical neurons were prepared as before^54^. All cells were maintained at 5% CO_2_ and 37 °C. To induce neuronal differentiation, SH-SY5Y cells were treated with 10 μΜ retinoic acid for 7 days and CAD cells were deprived of FBS for the same time period. Transfection of SK-N-SH and Neuro-2a cells was carried out using Lipofectamine 2000 (Invitrogen) following manufacturer’s recommendations. Co-transfections using APP minigene constructs and RBP expression plasmids were generally performed in a 1:10 ratio, unless otherwise stated. Transfected cells were harvested 48 hours after transfection.

### Whole cell lysate and nuclear extract preparation

Whole-cell lysates used for immunoblotting were prepared in a lysis buffer containing 20 mM Tris pH 7.5, 150 mM NaCl, 1 mM EDTA, 1%Triton X-100 and a protease inhibitor cocktail (Complete®, Roche). For RNA pull-down assays (see below), Neuro-2a cells were lysed in NT2 buffer (50 mM Tris pH 7.4, 150 mM NaCl, 1mMMgCl_2_, 0.05% NP40, protease inhibitor cocktail) supplemented with RNAse inhibitors (RNaseOUT, Invitrogen). In both cases, cellular debris was pelleted by centrifugation at 13,000 rpm for 10 min. In order to obtain nuclear extracts, cells were initially lysed in HLB buffer (10 mM Tris pH 7.4, 10 mMNaCl, 3 mM MgCl_2_, 1 mM EGTA, 0.1% NP40 and inhibitors for proteases and RNAses). Nuclei were pelleted by centrifugation at 2,500 rpm for 15 min, resuspended in RIP buffer (25 mM Tris pH 7.4, 150 mM KCl, 5 mM EDTA, 0.5% NP40 and protease and RNase inhibitors) and mechanically sheared using a dounce homogenizer. Nuclear membrane and debris were pelleted by centrifugation at 13,000 rpm for 10 min. Protein content in both whole cell lysates and nuclear extracts was determined by the Broadford assay (BioRad).

### Immunoblotting

Immunoblotting was carried out as previously described^54^. Briefly, equal amounts of whole cell extracts (30 μg) or equal volumes of pull-down materials were analyzed by 10% SDS-PAGE under reducing conditions. The molecular weight of proteins was confirmed using prestained protein ladder (PageRuler, Fermentas). The R1(57) anti-APP C-terminal rabbit antibody^55^, a kind gift of Dr. S. Efthimiopoulos (University of Athens, Greece) and the rabbit anti-AUF-1 antibody (07-260, Millipore) were used in a dilution 1:2000, the mouse anti-ELAVL and the rabbit anti-U2AF65 antibodies (3A2 and H300, respectively; all from Santa Cruz Biotechnology) in a dilution 1:1000 and the mouse anti-SAP97 (K64/15 UC Davis/NIH NeuroMab Facility and Antibodies Inc.) in a 1:50 dilution. Finally, the mouse anti-β-tubulin, the mouse anti-βΙΙΙ-tubulin (T5201 and T8660, respectively from Sigma) and the mouse anti-GAPDH-HRP conjugated (HRP-60004 from Proteintech) antibodies were used in a dilution 1:5000. All secondary HRP-conjugated antibodies (Santa Cruz Biotechnology) were used in a 1:5000 dilution. Each sample was tested in duplicate and samples obtained from three independent experiments were used for analysis. Densitometric analysis of immunoblotting images was performed using the image analysis software Image J (NIH, USA).

### RNA isolation and RT-PCR

Total RNA was extracted from cells using the RNAzol Reagent (Molecular Research Center, Inc) according to the manufacturer’s protocol, or from RIP material (see below) by acidic phenol/chlorophorm extraction. One microgram of RNA isolated from cells or total RNA isolated from RIP immunoprecipitates, was reverse transcribed by the M-MLV reverse transcriptase (Invitrogen) using random hexamer primers. Semi-quantitative PCRs were performed using Kappa Taq polymerase (Kappa Biosystems) and specific primers (see Supplementary Table S6). Amplification bands were quantified by densitometric analysis using the image analysis software Image J (NIH, USA). The effect of RBPs on APP RNA processing was calculated as a percentage exon(s) exclusion [(exon(s) exclusion/exon(s) exclusion + exons(s) inclusion)] averaged by three independent experiments. Quantitative real-time PCR (qPCR) was carried out in the Light Cycler 96 instrument (Roche) using Platinum Taq polymerase (Invitrogen), SYBR Green Reagent (Roche) and primers specific for either each APP isoform cDNA or APP genomic sequences; total APP cDNA and intronic GAPDH were used for normalization (see Supplementary Table S6). Specificity of amplification products was confirmed by their dissociation curves. Each sample was tested in triplicates and samples obtained from three to four independent experiments were used for analysis of relative expression by the comparative CT method.

### Ribonucleoprotein immunoprecipitation (RIP)

RIP analysis was performed as previously described^31^. Briefly, nuclear extracts were incubated for 4–6 hours at 4 °C with protein A/G Sepharose beads (Santa Cruz Biotechnology) coated with the mouse anti-ELAVL antibody or control mouse IgGs (Santa Cruz Biotechnology). RNA was isolated from both immunoprecipitates and their supernatants by acidic phenol/chlorophorm extraction and was further studied by semi-quantitative RT-PCR and RT-qPCR analysis, respectively. Binding of *APP* or *App* primary transcripts was verified by RT-PCR with primers specific for intronic regions of the pre-mRNA of each species, whereas binding specificity was ensured by the absence of GAPDH pre-mRNA, a known non target of nELAVs^27^, in immunoprecipitates (for primers see Supplementary Table S6).

### RNA pull-down assay

One microgram of linearized plasmid carrying the desired sequence was used as template for *in vitro* transcription with T7 RNA polymerase (Takara). Labeling of riboprobes was achieved with addition of biotin-11-CTP (Roche) in the nucleotide mix. Biotinylated probes were purified with acidic phenol/chlorophorm and used for RNA pull-down assays as previously described^56^. Briefly, 1 μg of biotinylated RNA was incubated with 100 μg of Neuro-2a whole extracts and RNA-proteins complexes were precipitated using streptavidin coated beads. For the detection of competitive binding, 0.4 μg of biotinylated RNA was incubated with 100 μg of whole cell lysate from transfected SK-N-SH cells and RNA-proteins complexes were precipitated as described above. To detect nELAVL-, AUF-1- or U2AF65- binding to the biotinylated transcripts, equal of volume of precipitates and their supernatants were analyzed by immunoblotting.

### Statistical analysis

Mean values were derived from at least three experiments. Depending on the number of groups examined, comparisons were carried out using either Student’s *t*-*test* (two groups) or one-Way ANOVA and Bonferroni *post*-*hoc* analysis (more than two groups). Significance was defined as P < 0.05. Statistical analysis was performed using the SPSS software (Release 10.0.1, SPSS, Chicago, IL, USA).

### Bioinformatics analysis

17 samples from 9 AD patient and 8 control post-mortem brain samples, subjected to NGS expression analyses and nELAVL HITS-CLIP, were retrieved from GEO repository (accession number GSE53699)^32^. Raw Seq data were initially quality checked with FastQC^57^ and further processed for adapter removal with a combined use of Minion^58^ and Trimgalore^59^. Following pre-processing, quantification of expression at gene and at transcript level was performed using Kallisto^60^. GRCh38 and Ensembl 81 were utilized as reference genomes and transcriptomes, respectively. All downstream analyses were implemented using R/Bioconductor^61^. The data had an average of 60 million PE reads. Initial standard differential expression analysis revealed that samples exhibited high heterogeneity, even within the same group. We found that this high level of intra-group variance diminished the number of differentially expressed transcripts between control and AD samples, an observation that was also noted by the authors of the original manuscript^32^. Subsequently, APP isoform levels were normalized against APP gene expression in each sample. Associations between normalized *APP* isoform expression and *Hu, TIA*-*1*, and *AUF*-*1* mRNA expression were investigated. Pearson Correlation Coefficient was calculated for all pairs to produce a correlation map. HITS-CLIP libraries had a mean value of ~10 M aligned reads. The adopted pipeline identified clusters of nELAVL binding corresponding to enriched regions with a 10 reads minimum coverage. The final set of nELAVL binding regions comprises peaks present in at least 2 samples.

### Figure preparation

Panels in figures were prepared from uncropped images deposited in Supplementary Material (Supplementary Figs S5–S12). For clarity reasons gel images were inverted and brightness and contrast adjustments were applied.

## Additional Information

**How to cite this article:** Fragkouli, A. *et al*. Neuronal ELAVL proteins utilize AUF-1 as a co-partner to induce neuron-specific alternative splicing of APP. *Sci. Rep.* **7**, 44507; doi: 10.1038/srep44507 (2017).

**Publisher's note:** Springer Nature remains neutral with regard to jurisdictional claims in published maps and institutional affiliations.

## Supplementary Material

Supplementary Information
