# Supplementary material for: Neuronal ELAVL proteins utilize AUF-1 as a co-partner to induce neuron-specific alternative splicing of APP
Source: Sci Rep. 2017 Mar 14;7:44507. doi: 10.1038/srep44507 (PMC5349543; doi:10.1038/srep44507)
Supplement: Supplementary Information [file srep44507-s1.doc]

**SUPPLEMENTARY MATERIALS**

**Neuronal ELAVL proteins utilize AUF-1 as a co-partner to induce neuron-specific alternative splicing of APP**

Apostolia Fragkouli, Pelagia Koukouraki, Ioannis S. Vlachos, Maria D. Paraskevopoulou, Artemis G. Hatzigeorgiou and Epaminondas Doxakis*

**SUPPLEMENTARY FIGURES**

**
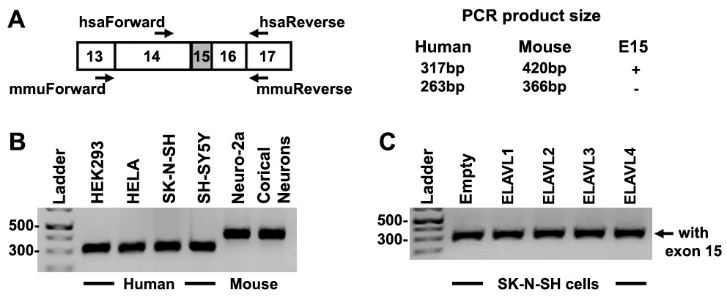
**

**Supplementary Figure S1. ELAVLs are not involved in the regulation of APP exon 15 AS.** (**A)** Schematic representation of exons 13 to 17 in the APP cDNA. Grey box depicts the alternative exon 15 and arrows the localization of human (hsa) and mouse (mmu) primers used for the simultaneous detection of the two transcript variants. PCR product size indicative of each transcript is shown. (**B**) Semi-quantitative RT-PCR was carried out using total RNA isolated from five cell lines and primary cortical neurons and the specific primers shown in panel A. Amplification bands, identified by their length, correspond to human and mouse transcripts containing APP exon 15. (**C**) Human SK-N-SH cells were transfected with the pCAGGS expression vector bearing either no insert (empty) or one of the ELAVL family members. The effect of ELAVs on the inclusion of APP exon 15 was assayed two days later by semi-quantitative RT-PCR, as described above. Note that overexpression of ELAVLs did not alter the AS pattern of APP exon 15.

**
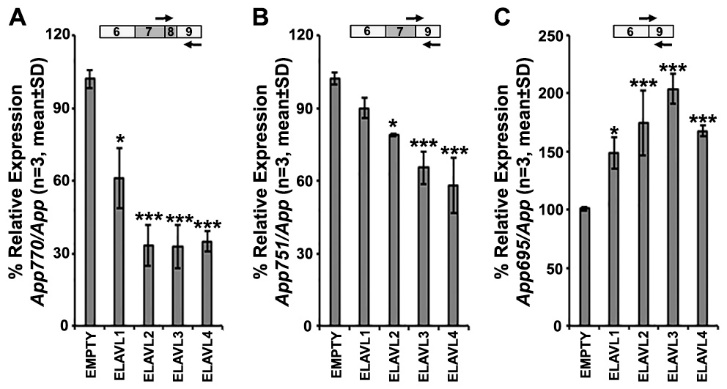
**

**Supplementary Figure S2. ELAVLs promote the simultaneous exclusion of exons 7 and 8 from the endogenous *App* pre-mRNA.** Mouse Neuro-2a cells were transfected with the pCAGGS expression vector bearing either no insert (control) or one of the four ELAVL members and the effect of ELAVLs on AS of *App* exons 7 and 8 was assayed two days later. Total RNA isolated from the transfected Neuro-2a cells was used in RT-qPCR experiments with primers specific for *App770* (**A**, *arrows*), *App751* (**B**, *arrows*) and *APP695* (**C**, *arrows*). In order to avoid confounding effects attributed to changes in *App* mRNA levels, total *App* cDNA was used for normalization. Bars in graphs correspond to mean ± standard deviation of three independent experiments. Statistical significance was determined by one-way ANOVA and Bonferroni post-hoc analysis (* P<0.05, *** P<0.001). Note that overexpression of all nELAVLs, but also of ELAVL1, upregulated selectively the relative expression of the *App695* transcript.

**
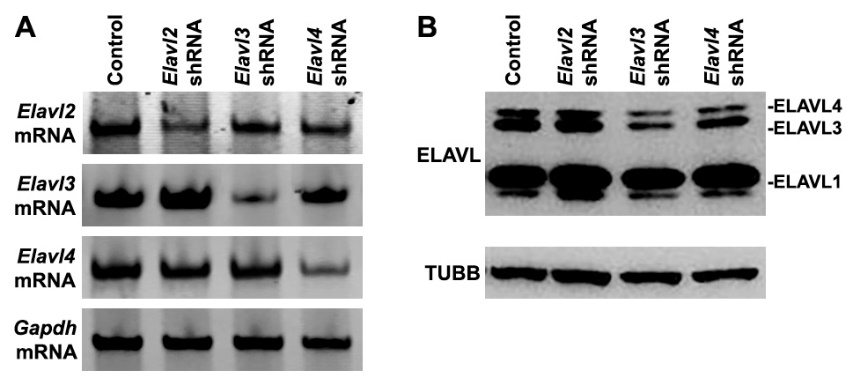
**

**Supplementary Figure S3.** Mouse Neuro-2a cells were transfected with an expression vector carrying a shRNA specific for eGFP *Elavl2*, *Elavl3* or *Elavl4* mRNA. Reduction of the expression of each nELAVL was determined two days post-transfection by RT-PCR (A) or immunoblotting (B) using total RNA or whole cells lysates, respectively.

**
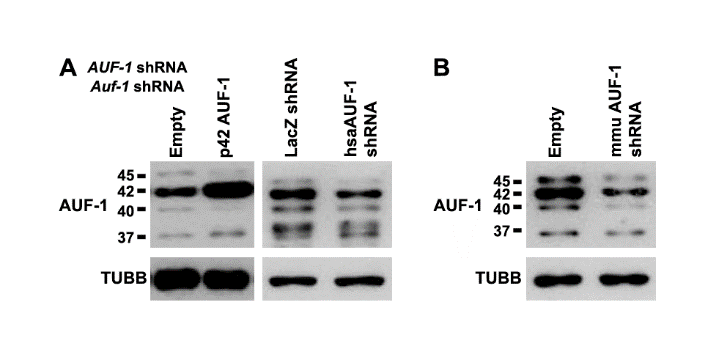
**

**Supplementary Figure S4.** (**A**) Human SK-N-SH cells were transfected with the DNA3.1 expression vector bearing either no insert (empty) or p42 AUF-1, as well as with the pENTR/U6 vector carrying a shRNA specific for LacZ or all *AUF-1* mRNAs. (**B**) Mouse Neuro2a cells were transfected with the pSilencer vector bearing either no insert (empty) or a shRNA targeting all *Auf-1* transcripts. To verify changes in AUF-1 expression, equal amounts of total protein from lysates of the transfected SK-N-SH (A) and Neuro2a (B) cells were analyzed on 10% SDS-PAGE and immunoblotted with an antibody against AUF-1; membranes were also probed against β-TUBULIN (TUBB) in order to ensure equal loading.

**
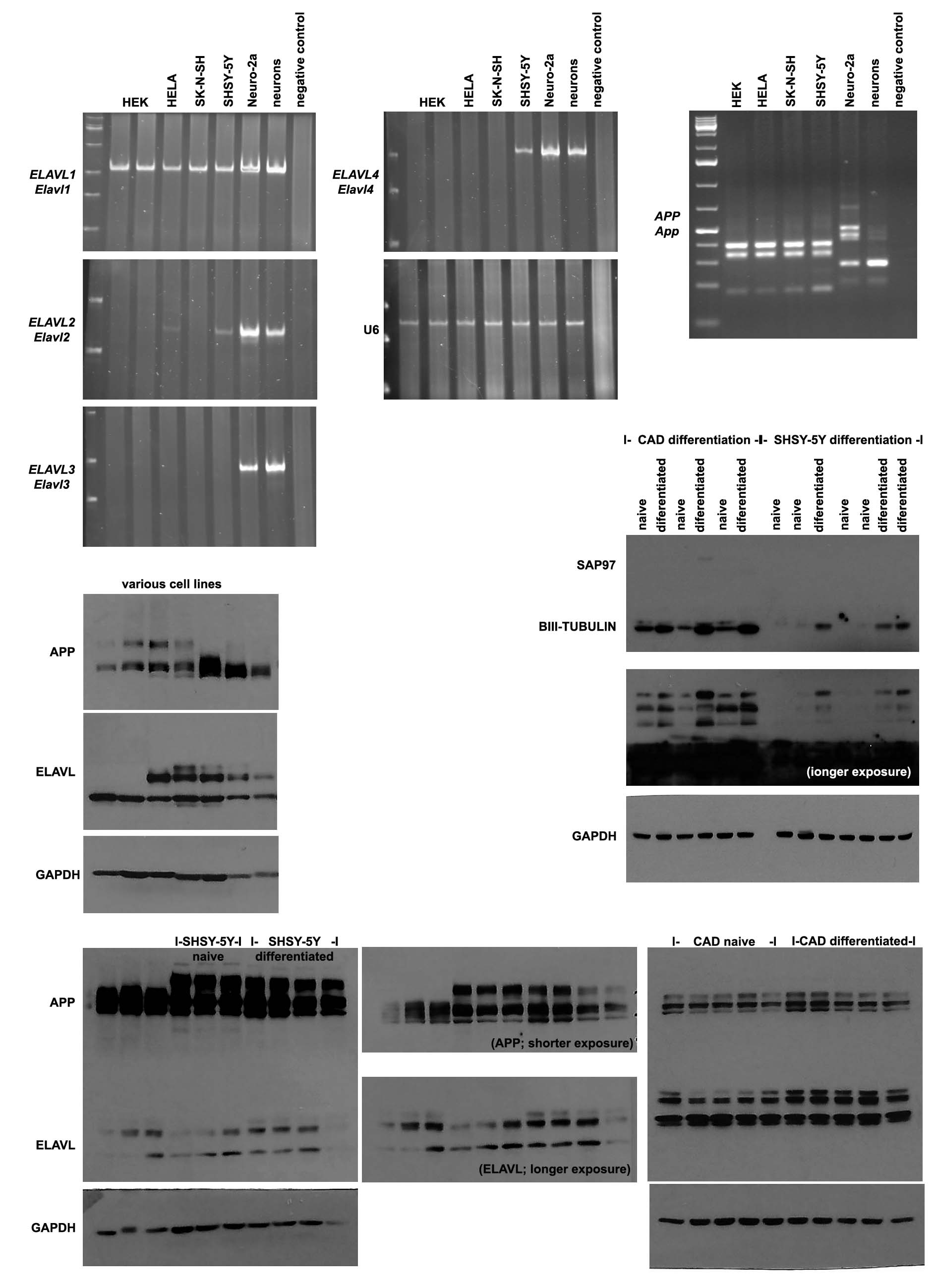
**

**Supplementary Figure S5.** Original images used for Figure 1

**
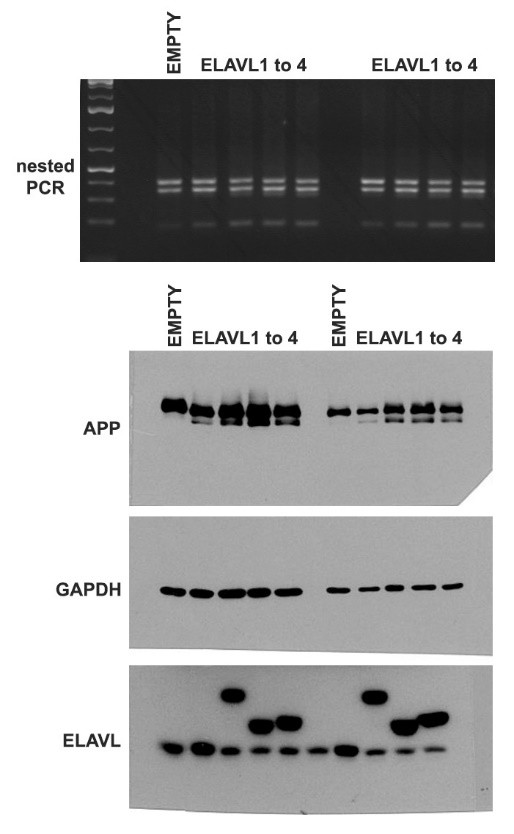
**

**Supplementary Figure S6.** Original images used for Figure 2


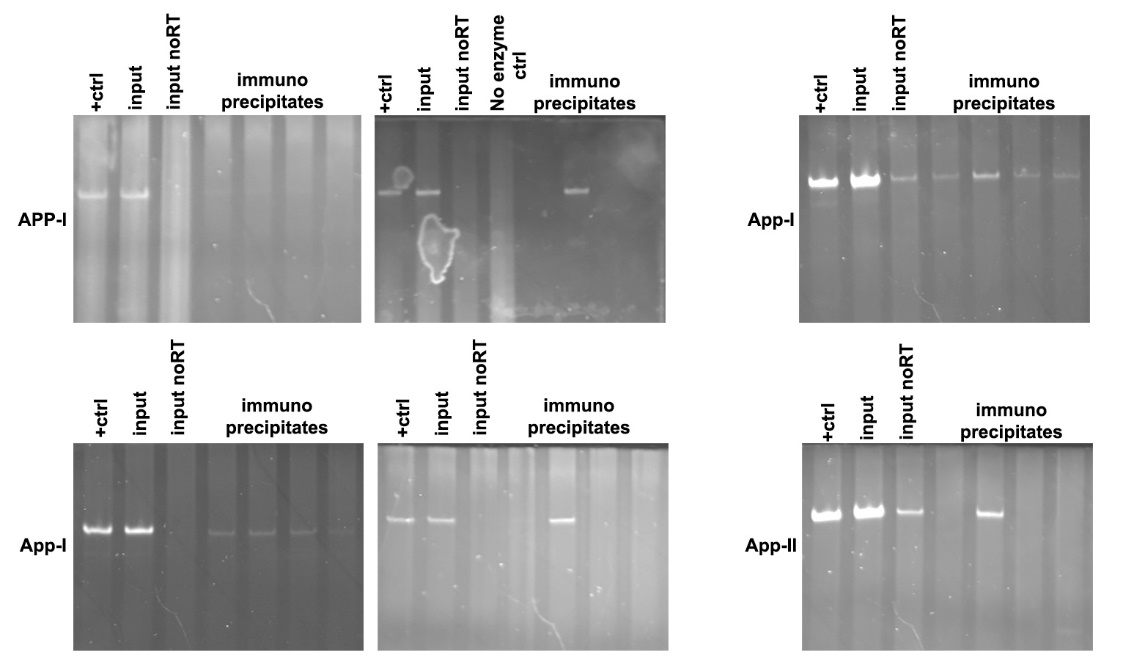


**Supplementary Figure S7.** Original images used for Figure 3


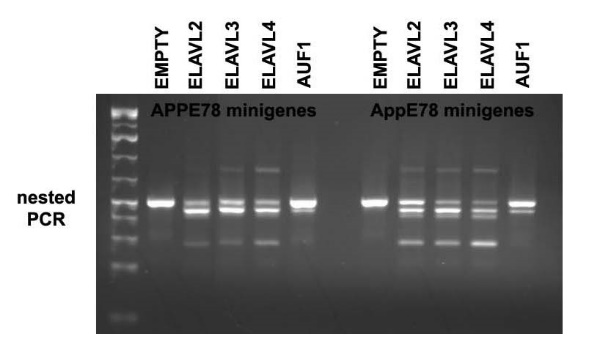


**Supplementary Figure S8.** Original images used for Figure 4


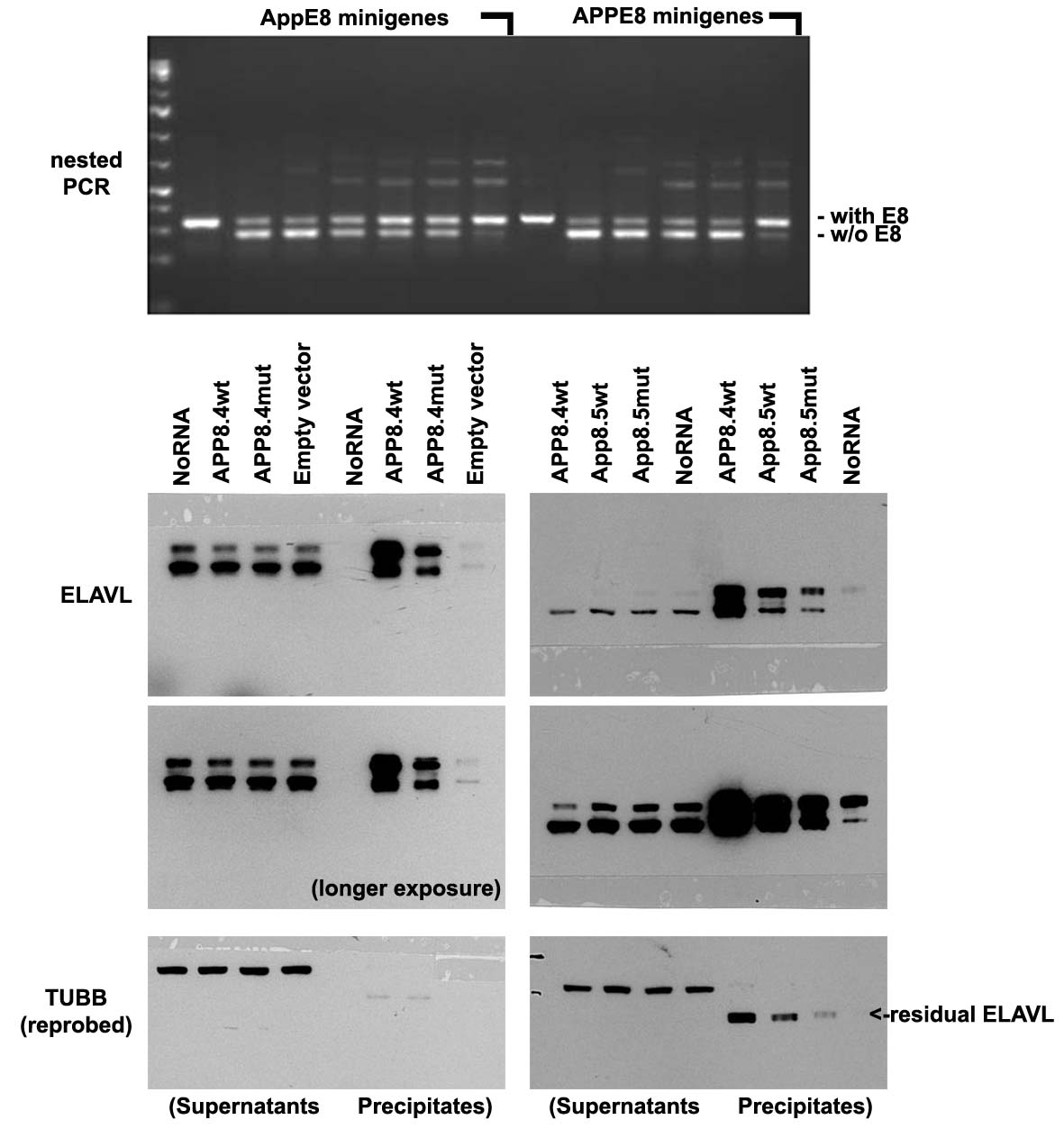


**Supplementary Figure S9.** Original images used for Figure 5


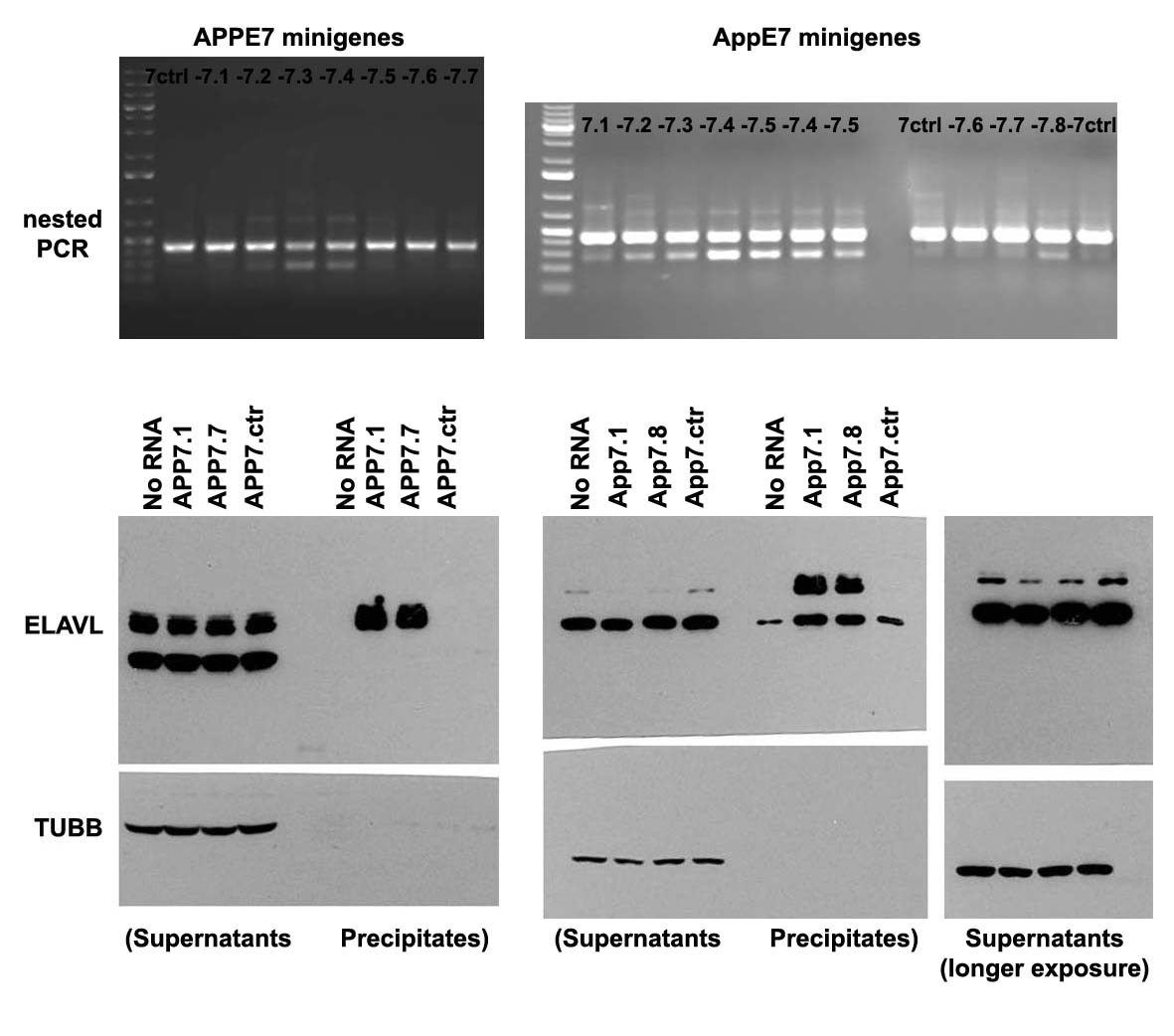


**Supplementary Figure 10.** Original images used for Figure 6


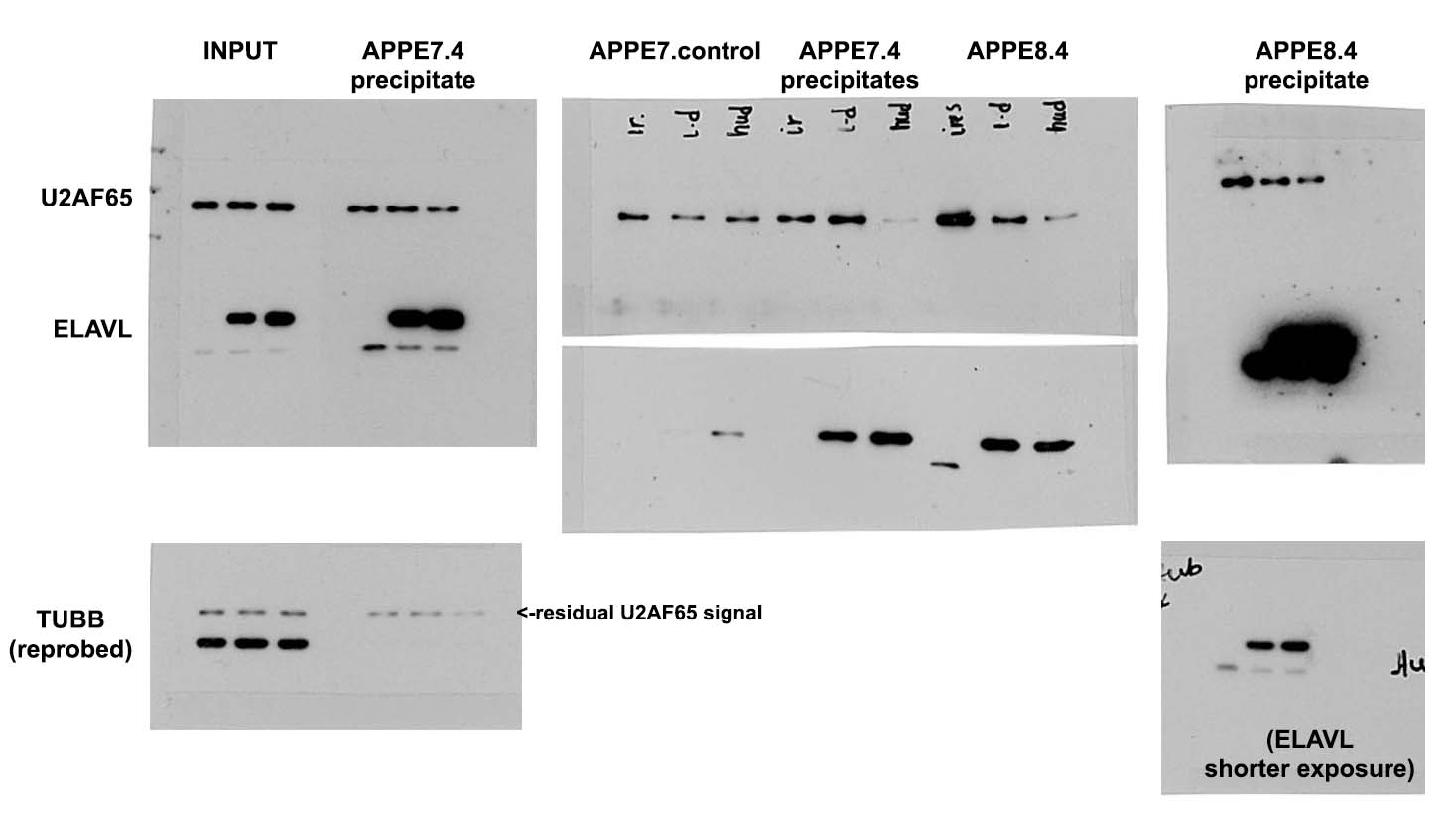


**Supplementary Figure 11.** Original images used for Figure 7


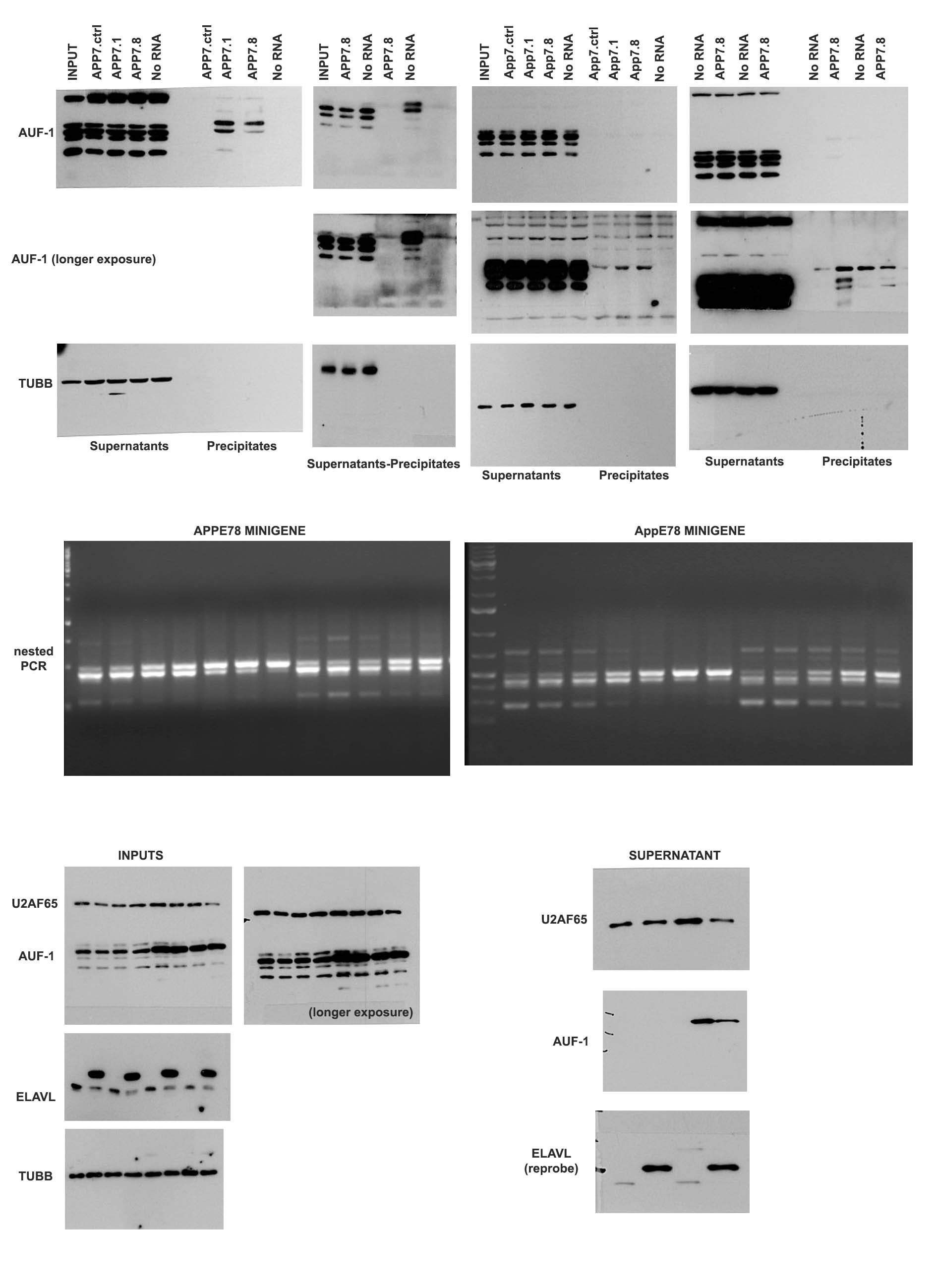


**Supplementary Figure 12.** Original images used for Figure 8

**SUPPLEMENTARY TABLES**

**Supplementary Table S1. Correlation between APP isoform levels and RBP mRNA levels (A)** Correlation coefficient **(B)** P value<0.05

| **A.** | **APP770** | **APP695** | **APP751** | **APP** | **ELAVL1** | **ELAVL2** | **ELAVL3** | **ELAVL4** | **AUF1** | **TIA1** |
| --- | --- | --- | --- | --- | --- | --- | --- | --- | --- | --- |
| **APP770** | 1 | 0.120 | -0,088 | -0.338 | -0.267 | -0.252 | -0.167 | -0.382 | 0.439 | 0.223 |
| **APP695** | 0.120 | 1 | -0.316 | 0.583 | 0.172 | 0.593 | -0.510 | 0.544 | 0.370 | 0.047 |
| **APP751** | -0.088 | -0.316 | 1 | 0.316 | 0.576 | -0.059 | -0.238 | 0.051 | -0.571 | 0.152 |
| **APP** | -0.338 | 0.583 | 0.316 | 1 | 0.314 | 0.694 | -0.404 | 0.735 | -0.042 | 0.245 |
| **ELAVL1** | -0.267 | 0.172 | 0.576 | 0.314 | 1 | 0.172 | -0.279 | 0.235 | -0.554 | -0.265 |
| **ELAVL2** | -0.252 | 0.593 | -0.059 | 0.694 | 0.172 | 1 | -0.336 | 0.917 | 0.336 | 0.240 |
| **ELAVL3** | -0.167 | -0.510 | -0.238 | -0.404 | -0.279 | -0.336 | 1 | -0.333 | -0.039 | -0.132 |
| **ELAVL4** | -0.382 | 0.544 | 0.051 | 0.735 | 0.235 | 0.917 | -0.333 | 1 | 0.211 | 0.206 |
| **AUF1** | 0.439 | 0.370 | -0.571 | -0.042 | -0.554 | 0.336 | -0.039 | 0.211 | 1 | 0.507 |
| **TIA1** | 0.223 | 0.047 | 0.152 | 0.245 | -0.265 | 0.240 | -0.132 | 0.206 | 0.507 | 1 |

| **B.** | **APP770** | **APP695** | **APP751** | **APP** | **ELAVL1** | **ELAVL2** | **ELAVL3** | **ELAVL4** | **AUF1** | **TIA1** |
| --- | --- | --- | --- | --- | --- | --- | --- | --- | --- | --- |
| **APP770** | - | 0.646 | 0.736 | 0.184 | 0.300 | 0.328 | 0.523 | 0.130 | 0.078 | 0.390 |
| **APP695** | 0.646 | - | 0.216 | 0.014 | 0.510 | 0.012 | 0.037 | 0.024 | 0.144 | 0.859 |
| **APP751** | 0.736 | 0.216 | - | 0.216 | 0.016 | 0.823 | 0.358 | 0.844 | 0.017 | 0.560 |
| **APP** | 0.184 | 0.014 | 0.216 | - | 0.220 | 0.002 | 0.107 | 0.001 | 0.874 | 0.343 |
| **ELAVL1** | 0.300 | 0.510 | 0.016 | 0.220 | - | 0.510 | 0.277 | 0.363 | 0.021 | 0.305 |
| **ELAVL2** | 0.328 | 0.012 | 0.823 | 0.002 | 0.510 | - | 0.188 | 0.000 | 0.188 | 0.353 |
| **ELAVL3** | 0.523 | 0.037 | 0.358 | 0.107 | 0.277 | 0.188 | - | 0.191 | 0.881 | 0.613 |
| **ELAVL4** | 0.130 | 0.024 | 0.844 | 0.001 | 0.363 | 0.000 | 0.191 | - | 0.417 | 0.428 |
| **AUF1** | 0.078 | 0.144 | 0.017 | 0.874 | 0.021 | 0.188 | 0.881 | 0.417 | - | 0.038 |
| **TIA1** | 0.390 | 0.859 | 0.560 | 0.343 | 0.305 | 0.353 | 0.613 | 0.428 | 0.038 | - |

**Supplementary Table S2: Human genomic sequence (Ensembl: ENSG00000142192) used for the generation of *APPE78* minigene.** Sequences encoding for U-rich blocks in the pre-mRNA are underlined.

| **INTRON 6** (total length: 21658bp) | |
| --- | --- |
| …....21200  21201- 300  21301- 400  21401- 500  21501- 600  21601- 658 | ……………………………………………………………caacactggaaaacattcaaaatatatgcaattaattttata  tttgagttaaggcaatcatttattaaggaaagcaatcagatgtttgtgagggcttaaactttcgcttcataaccaaagcagtttccttacatgaaagttg  acttcttaaaaagagaagtttattttttatgatggaggattgttgtgatgtgcagataccttccgtcatttcatactcatctaaaagcagccaaatgatc  ttttcacctttggccggaatccttttattgctaccgagtcctgcactcattaaaccagatgacagtgcatgctctgagaggtgtaaatctctagtcctgg  tggccagttaaattcctcagtaaatgtttggtagatgctgcctaataaaccagtccaggttgccactgggaggattaaaagaagtaaacgtgtatacatg  aacagagagacagtgccttttcatgctaaatgtggttccccacatctcctctgattag |
| **EXON 7** (total length: 168bp) | |
| **AGGTGTGCTCTGAACAAGCCGAGACGGGGCCGTGCCGAGCAATGATCTCCCGCTGGTACTTTGATGTGACTGAAGGGAAGTGTGCCCCATTCTTTTACGGCGGATGTGGCGGCAACCGGAACAACTTTGACACAGAAGAGTACTGCATGGCCGTGTGTGGCAGCGCCA** | |
| **INTRON 7** (total length: 2598bp) | |
| 1…700  701- 800  801- 900  900....200  1201- 1300  1301- 1400  1401..1600  1601- 1700  1701- 1800  1801- 1900  1901- 2000  2001- 2400  2401 -2500  2501 -2598 | gtaagtggacccttcttcgagcctggccac…………………………………….ctttcaatgacataaggctcgaggaacata  attttattttttttgtttttttaaagacagggtctcattctgtctcacaggctggagtgcaatggcgtgatcttggctcactgcaacctccgcctcccgg  gttcaagtgattctcctgcctcagcctccccagcagctaggatcacaggcacatgccactgcgcccggctaattttttgtatttttggtagagacaaggc  ttcaccatgttgaccaggctggtctcgaac………........................................................acttccaagtgaaattcccaattgtgctta  tttatttatttatttatttatttgttgttgttgttcatgagtaggaagtatgaactactcaagaagtaattattcagctcagcttgataactaaaaatga  aactcaataactgtgtattacttttttgagagactcctgcctaattctccgctgtgttgcaagggtggcagccacatttcctgcctgagattcctaggga  ttatataaattgttttgcagcaagatctta………......................................................tgtgtgaacaggtgtaggaactgataacct  gaagggctttttttttttttttttctggctctgattggatgattgcagcttctggatcaaaggactcccttcttcagtttcaaacaaagcagtcaattag  gtcaggtattgcgaacacaatggagaattctgttgcctgcagcctttggaacaaaggccttttgttacagtgattctcactggtttagccggtgggagag  gtttcctggcagatttgcaacttttttctccctttccctgggtggattcattttctgaaatatcatgtatagtttttggtaataagagatttatttggtt  ttaattattagcagtgatgaatttgcttcaagagattaaaattctctccacctcaccccagtactgccatcccttcacctaacactctctccctttgatc  agtttgggctgggagtgaccagtgggcacc……………………………………..actcggatatactatcagagacaaaatatc  aagcattctcaaatgttaacttcttacgaaaatagatcttatgtttatatgttcattttggttttgttggagggaccaaacctaagtgagtgattttgtt  tgttaggttgtttttttgtcagtggactcgtgcatttcagccatcattcccatgtttctctttttgtttttagttatgttctcttattttttccatag |
| **EXON 8** (total length: 57bp) | |
| **TGTCCCAAAGTTTACTCAAGACTACCCAGGAACCTCTTGCCCGAGATCCTGTTAAAC** | |
| **INTRON 8** (total length: 14884bp) | |
| 0-100  101-200  201-300  301-400  401-500  501- ..... | Gtacgttgtcattcacctgagggaagggaagaggggaggaggatgctgcttggttcacataactccagcatcatcaccttctttgcatggttttgtgttt  cttgaacacctgtcttagtaaaatgtttcttcccattaccttgcttgtaattacatctgattttgccagacagcttgagatgttgggctaagagcatcat  tgactaagtttcttctatttctgaccaatttcctttttatttagtctggttttattgaatattatatggacaacatcattgtattgtatttgccattact  attttatttcctaaaagctatcagtgtaactgagagcaggcttagcctctcactgcttttgcagaactgaagaacaagggctaggtgcagtggaaggaaa  gtgactttacttagcaaagctagcaatggggaaatggtccaggctcctgcctcaaagcaaccatctcaaattttggattaaaaaacaaaggcttaaaaag  gggag... |

**Supplementary Table S3: Mouse genomic sequence (ENSMUSG00000022892) used for the generation of *AppE78* minigene.** Sequences encoding for U-rich blocks in the pre-mRNA are underlined.

| **INTRON 6** (total length: 12580bp) | |
| --- | --- |
| ....... 12000  12101- 200  12201- 300  12301- 400  12401- 500  12501- 580 | ………………………………………………………………………………………….acattaaacataaatgtga  ttacttatgcatgagtctaggcgatcatttactaagaaaaacagtcagaagttggagagaacgtaaattttggcatcacatcccgagaaatccttttttt  ttttttttttttttaaacatcagagttgactcaaggttgttttagaagatgccttttttttttaagagaaatttttgatgtgcagagagagagacttttt  atctttaaattgagaaaacaaccttttcacttctagcccaaatcctgtagctgctactgcatcctacacattaaacaagccgagaatgtcttgttttgtg  gaagaggtgactctgaagtctgagtgggtcaattcagtccctcaaggaagacttaaagtagctgcttcctccgttcagcccaggtcaccactgggaggat  cagggcttaagttgagtaagtatgtatgtgaacagccagaagatgtcttttctcgctaaatgtggttcccctctgattag |
| **EXON 7** (total length: 168bp) | |
| **AGGTGTGCTCTGAACAAGCCGAGACCGGGCCATGCCGCGCAATGATCTCCCGCTGGTACTTTGATGTCACTGAAGGGAAGTGTGTCCCATTCTTTTACGGCGGATGTGGCGGCAACAGGAACAACTTTGACACGGAAGAGTACTGCATGGCGGTGTGTGGCAGCGTGT** | |
| **INTRON 7** (total length: 3268bp) | |
| 0-500  501-900  901-1000  1001-1500  1501-1600  1601-1700  1700-2100  2101-2200  2201-3000  3001-3100  3101-3200  3201-3268 | gtaagtggatccttcctccagcctggcc……………………………………....ttcctcttatttcatcatggttttttttttttttaac  ttcattctgataatgacatttaattgaat……………………………………....tctgtaggggcctatgtggcccattttcattcaa  accattatactatgggtgcccatgctgttccctgaatgaacctcgagcacatattcttgccaagtttttctttttttctcatgcaattaggtagacgaga  aatagggatcgggaccatttaataaaa……………………………...………..atgtctcagatatatggaatatctgcttctatta  gtagcctttcaagcaaagtccttagttgtatttgtttattcttgatcacaagttggctatatgaattattcaagggtcttagtgacagtgcatgcctttc  ttgagagattcttgcattttaattcttgaatttgcagggagacatatcccttccctttccctggatatctgggaacattctggcaattttccaaccaaat  cccagaaccaagcaaaacaaagactt……………………………………..agggttagtttctggaatatcatattctagcgatc  aggaagtttgctttatattattagcagagaacaattcaactcaagttttggacactgtcttcttacccaccctagtgtaaacccccctcatctcccttcc  acttgattactctgtgcagggagtata………………………………………cccctaagtcctcctgtagatttcccagtgtcctg  tcagtggcagaatgggcagcaatttcacaatactgatctttactggtaacaaatctcacctgcatttccttggttctttgtttaggttttaattttagtt  tcatgatttccctcccagccctcaattttgtttgattgcttttgagtcctgtgggtgtgtttctgccatcattcccacctttctgttgttggggtttttt  tttgtttgtttgtttgttttgttttgttttgttttgtttttagttatgttctctcgttttctccatag |
| **EXON 8** (total length: 57bp) | |
| **CAACCCAAAGTTTACTCAAGACTACCAGTGAACCTCTTCCCCAAGATCCTGATAAAC** | |
| **INTRON 8** (total length: 9885bp) | |
| 0-100  101-200  201-300  301-400  401-….. | gtatgttgtcactgacttgggggaggggcaaggggaagactatgttgcatgactgaatgtgtccttcccttgcatcatctgcttggcatgggttttgtgt  gattattgaccaggaaccttttgatagatactattttcccattgtcttgcgtgtcatttcatccggtttttactaggcaacttcagatgttgggcagaga  gcatctacttaaaccatttacacttctgaacaaattcctttctgatttgtgttgttctgtgaaataccacacatagagtatttcttgatttggcagaact  tttttttttaatttcccgaaagctgtcaacatgccataatttaatgaaatcatatttgatagtacaggtaaccccctccctaaaaatgtaatattgctta  ttcaagacaattttgtgtgcacatttcaaggttcctgtgttaaatgataactgtgcagcattgggaggtgaaaggtgagccacatag |

**Supplementary Table S4: Genomic locations of nELAVL binding sites on human APP transcript.** nELAVL peaks depicted in Figure 5A are present in at least 2 HITS-CLIP libraries derived from AD patient and control post-mortem brain samples.

| **chromosome** | **Start** | **end** | **cluster** | **width** |
| --- | --- | --- | --- | --- |
| chr21 | 25997091 | 25997142 | nELAVL1 | 52 |
| chr21 | 25997401 | 25997460 | nELAVL2 | 60 |
| chr21 | 25997497 | 25997532 | nELAVL3 | 36 |
| chr21 | 25997735 | 25997805 | nELAVL4 | 71 |
| chr21 | 25998376 | 25998421 | nELAVL5 | 46 |
| chr21 | 25998740 | 25998834 | nELAVL6 | 95 |
| chr21 | 25998879 | 25998924 | nELAVL7 | 46 |

**Supplementary Table S5. Oligonucleotides used as primers for cloning cDNA and genomic sequences**

| **Oligonucleotide** | **Sequence** |
| --- | --- |
| **mmuELAVL1_5´** | GGTACCGCCGCCATGTCTAATGGTTATGAAGAC |
| **mmuELAVL1_3´** | GCGGCCGCAGCATGAGCGAGTTATTTGTGGGACTTGT |
| **mmuELAVL2_5´** | GGTACCGCCGCCAGGAAACACAACTGTCTAATGGG |
| **mmuELAVL2_3´** | GCGGCCGCACTGAGGACAAGAGCTCATTAGGCTTTGT |
| **mmuELAVL3_5´** | GGTACCGCCGCCATGGTCACTCAGATACTGGGG |
| **mmuELAVL3_3´** | GCGGCCGCAATGCTCAGGCCTTGTGCTGCTTG |
| **mmuELAVL4_5´** | GGTACCGCCGCCATGGAGTGGAATGGCTTGAAGATGA |
| **mmuELAVL4_3´** | GCGGCCGCTAATAAGTAAGGGTGAGAAAT |
| **hsaAUF-1 shRNA_5´** | CACCGCCTGAATGGAAGTATGACGTTCTCGAGAACGTCATACTTCCATTCAGG |
| **hsaAUF-1 shRNA_3´** | CGGACTTACCTTCATACTGCAAGAGCTCTTGCAGTATGAAGGTAAGTCCAAAA |
| **SD_F** | TTTCGCCAACGGGTTTGC |
| **SD_R** | CGCAGCTAATCCCCGCCGAC |
| **SA_F** | ACAGGATCAAGGGATCTGTAGGGC |
| **SA_R** | ACACTCGAGATCCACTAGTTCTAGA |
| **APP7_514F** | ATTGCGGCCGCGGACAGACAGCTTCCAACA |
| **APP7_51F** | ATTGCGGCCGCAGACAGTGCCTTTTCATGCTAA |
| **APP7_2112R** | ACGGTCGACCTCATCTAATACATCTCTGT |
| **APP7_1603R** | ACGGTCGACTTGAGGTTATCAGTTCCTACACC |
| **APP7_1078R** | ACGGTCGACACTTCATATTTTTATCCCCCG |
| **APP7_525R** | ACGGTCGACTCACATTCCCTCATCAGCACAA |
| **APP7_83R** | ACGGTCGACCAGGAAAATCAATCTGTTACAA |
| **App7_512F** | ATTGCGGCCGCGGACAGGCTGGAAACATTA |
| **App7_72F** | ATTGCGGCCGCAAGTTGAGTAAGTATGTATGT |
| **App7_2799R** | ACGGTCGACCTATTCTTCCCTTTCCTTCT |
| **App7_1766R** | ACGGTCGACCAGATATTAGCTACCTCACAG |
| **App7_1014R** | ACGGTCGACTCCCGATCCCTATTTCTCGT |
| **App7_515R** | ACGGTCGACCATTATCAGAATGAAGTTAA |
| **App7_70R** | ACGGTCGACCTGTTACAAAGAGCAGAGCC |
| **APP8_506F** | ATTGCGGCCGCACAGAGATGTATTAGATGAG |
| **APP8_164F** | ATTGCGGCCGCGATCTTATGTTTATATGTTCATT |
| **APP8_69F** | ATTGCGGCCGCGTGCATTTCAGCCATCATTC |
| **APP8_47F** | ATTGCGGCCGCATGTTTCTCTTTTTGTTTTTAG |
| **APP8_47mutF** | ATTGCGGCCGCATGTCTCTCTTGTTGTACTTAG |
| **APP8_505R** | ACGGTCGACCTCCCCTTTTTAAGCCTTTGT |
| **APP8_208R** | ACGGTCGACCTTAGTCAATGATGCTCTTAGCC |
| **APP8_82R** | ACGGTCGACAGAAGGTGATGATGCTGGAGTT |
| **App8_489F** | ATTGCGGCCGCAGAAGGAAAGGGAAGAATAG |
| **App8_208F** | ATTGCGGCCGCTGCATTTCCTTGGTTCTTTGT |
| **App8_113F** | ATTGCGGCCGCTGTGTTTCTGCCATCATTCC |
| **App8_89F** | ATTGCGGCCGCTTTCTGTTGTTGGGGTTTTTTT |
| **App8_47F** | ATTGCGGCCGCTTTTGTTTTGTTTTGTTTTTA |
| **App8_47mutF** | ATTGCGGCCGCTTCTGTTATGTTCTGTTGTTA |
| **App8_487R** | ACGGTCGACTATGTGGCTCACCTTTCACCT |
| **App8_278R** | ACGGTCGACCTCTATGTGTGGTATTTCACAG |
| **App8_162R** | ACGGTCGACATGAAATGACACGCAAGACAATG |
| **App8_77R** | ACGGTCGACTGATGCAAGGGAAGGACACATT |

Supplementary Table S6. Oligonucleotides used as primers for semi-quantitative (*) and real-time (#) PCR

| **Oligonucleotides** | **Sequence** |
| --- | --- |
| **hsa/mmuELAVL1_F*** | GGCTGGTGCATCTTCATCTA |
| **hsa/mmuELAVL1_R*** | CACGAATCACTTTCACATTGG |
| **hsa/mmuELAVL2_F*** | ATACCGCCTGGGAGACAGAG |
| **hsa/mmuELAVL2_R*** | AATGGACTGAGGACAAGAGC |
| **hsa/mmuELAVL3_F*** | CTGTCGCTCATCGCCAGGTTC |
| **hsa/mmuELAVL3_R*** | CAGCACGCTCTCATCAGCTTC |
| **hsa/mmuELAVL4_F*** | CAATACGGTCGCATCATCAC |
| **hsa/mmuELAVL4_R*** | TGGCTTCTTCTGCCTCAATC |
| **hsa/mmuU6_F*** | CGCTTCGGCAGCACATATAC |
| **hsa/mmuU6_R*** | TTCACGAATTTGCGTGTCAT |
| **mmuGAPDH_F*** | TCCAGTATGACTCCACTCAC |
| **mmuGAPDH_R*** | TCCTGGAAGATGGTGATGG |
| **hsaAPPEx6_F*** | GAAGAGGCTGAGGAACCCTACG |
| **hsaAPPEx9_R*** | CATGTTCATTCTCATCCCCAGG |
| **hsaAPPE14_F*** | CGATGCTCTCATGCCATCTTT |
| **hsaAPPE17_R*** | CCCACATCTTCTGCAAAGAAC |
| **mmuAPPEx6_F*** | GTAGTAGAAGTCGCCGAAGAGGA |
| **mmuAPPEx9_R*** | CTTTCTGGAAATGGGCATGCTC |
| **mmuAPPE13_F*** | GAGGAGATTCAAGATGAAGTCG |
| **mmuAPPE17_R*** | CCCACATCTTCAGCAAAGAAC |
| **AEI_F*** | GCTGGCTAGTTAAGCTATCAAC |
| **AEII_R*** | CCTCTAGATCAACCACTTTGTAC |
| **hsaAPP770_F#** | GTGGCAGCGCCATGTCCCAA |
| **hsaAPP751_F#** | GGCAGCGCCATTCCTACAAC |
| **hsaAPP695_F#** | GGTGGTTCGAGTTCCTACAAC |
| **hsaAPPE9_R#** | TCTCTCGGTGCTTGGCCTCA |
| **mmuAPP770_F#** | TGTGGCAGCGTGTCAACCCA |
| **mmuAPP751_F#** | GGCAGCGTGTTTCCCACGAC |
| **mmuAPP695_F#** | TGGTCCGAGTTCCCACGACA |
| **mmuAPPE9_R#** | GACATTCTCTCTCGGTGCTTG |
| **hsa/mmuAPPE3/4_F#** | GCCAAAGAGACATGCAGTGAGA |
| **hsa/mmuAPPE5_R#** | GAGTCATCCTCCTCCGCATC |
| **hsa/mmuAPPE7_F#*** | CATTCTTTTACGGCGGATGTGG |
| **hsaAPPIn7_R#*** | TAGTGGTAGCAACAGGCCCAT |
| **mmuAPPIn7_R#*** | CCAGTGGCGAGAAAGACAAGAG |
| **hsaAPPIn7_F#*** | TCGTGCATTTCAGCCATCATT |
| **mmuAPPIn7_F#*** | GTTTCTGCCATCATTCCCACC |
| **hsa/mmuAPPE8_R#*** | GGTAGTCTTGAGTAAACTTTGGG |
| **hsaInGAPDH_F#*** | CGCGTCTACGAGCCTTGCGGCT |
| **hsaInGAPDH_R#*** | GCTTTCCTAACGGCTGCCCATTCA |
| **mmuInGAPDH_F#*** | AGGGCTCATGGTATGTAGGCA |
| **mmuInGAPDH_R#*** | TGGACTGTGGTCTAGAAAACACG |
